# EZH2-dependent phenotypic plasticity reveals TTP-associated and mTORC1 therapeutic vulnerabilities in lethal prostate cancer

**DOI:** 10.1016/j.xcrm.2026.103083

**Published:** 2026-09-30

**Authors:** Beatriz German, Katherine L. Morel, Teia Noel, Nadia Boufaied, Deborah L. Burkhart, Sujun Chen, Felipe Dezem, Xintao Qiu, Henry W. Long, Kun-Lin Ho, Stefan DiFazio, Sylvan Baca, Ayesha A. Shafi, Matthew L. Freedman, Himisha Beltran, Christopher J. Sweeney, Housheng Hansen He, Myles Brown, Jasmine T. Plummer, Simon.R.V. Knott, David P. Labbé, Leigh Ellis

**Affiliations:** 1 Center for Prostate Disease Research, Murtha Cancer Center Research Program, Department of Surgery, Uniformed Services University of the Health Sciences, Bethesda, MD, USA; 2 The Henry M. Jackson Foundation for the Advancement of Military Medicine, Inc., Bethesda, MD, USA; 3 Genitourinary Malignancies Branch, Center for Cancer Research, National Cancer Institute, Bethesda, MD, USA; 4 South Australian Immunogenomics Cancer Institute, University of Adelaide, Adelaide, SA, Australia; 5 Cedars-Sinai Center for Bioinformatics and Functional Genomics, Los Angeles, CA, USA; 6 Cancer Research Program, Research Institute of the McGill University Health Centre, Montréal, QC, Canada; 7 Department of Cancer Biology, Dana-Farber Cancer Institute, Harvard Medical School, Boston, MA, USA; 8 West China School of Public Health, West China Fourth Hospital, and State Key Laboratory of Biotherapy, Sichuan University, Chengdu, China; 9 Department of Developmental Neurobiology, St. Jude Children’s Research Hospital, Memphis, TN, USA; 10 Center for Functional Cancer Epigenetics, Dana-Farber Cancer Institute, Boston, MA, USA; 11 Department of Medical Oncology, Dana-Farber Cancer Institute, Harvard Medical School, Boston, MA, USA; 12 Princess Margaret Cancer Centre, University Health Network, Toronto, ON, Canada; 13 Department of Medical Biophysics, University of Toronto, Toronto, ON, Canada; 14 Department of Biomedical Sciences, Cedars-Sinai Medical Center, Los Angeles, CA, USA; 15 Division of Urology, Department of Surgery, McGill University, Montréal, QC, Canada

**Keywords:** EZH2, PI3K, mTOR, phenotypic plasticity, translation, prostate cancer

## Abstract

Phenotypic plasticity is a recognized mechanism of therapeutic resistance in prostate cancer (PCa); however, current knowledge of its underlying drivers and therapeutic interventions remain limited. Using genetically engineered mouse models (GEMMs) devoid of *Pten* and *Rb1*, we previously demonstrated the chromatin reprogramming factor enhancer of zeste homolog 2 (EZH2) as a regulator of alternative transcription programs promoting phenotypic plasticity. Here, using a multi-omics approach, we demonstrate that EZH2 regulates multilineage cell states and identify the RNA-binding protein Tristetraprolin (TTP) as a contributor to the response to EZH2 therapy through its role in RNA stability and translational regulation. Combined pharmacological inhibition of EZH2 and PI3K/mTORC1 produces superior antitumor activity in murine and human models of phenotypic plasticity, with greatest efficacy observed when combined with castration. Together, these findings identify EZH2-PI3K/mTORC1 signaling as a therapeutic vulnerability in phenotypically plastic PCa and reveal TTP-associated regulatory programs as contributors to targeted therapy response.

## Highlights

• EZH2 drives multilineage plasticity in RB1-deficient prostate cancer
• Multi-omics identifies TTP-associated programs linked to EZH2 inhibitor response
• EZH2 inhibition induces PI3K/mTORC1 signaling as a targetable vulnerability
• Combined EZH2 and PI3K/mTORC1 inhibition enhances antitumor activity with ADT

## Graphical abstract

**Figure:**
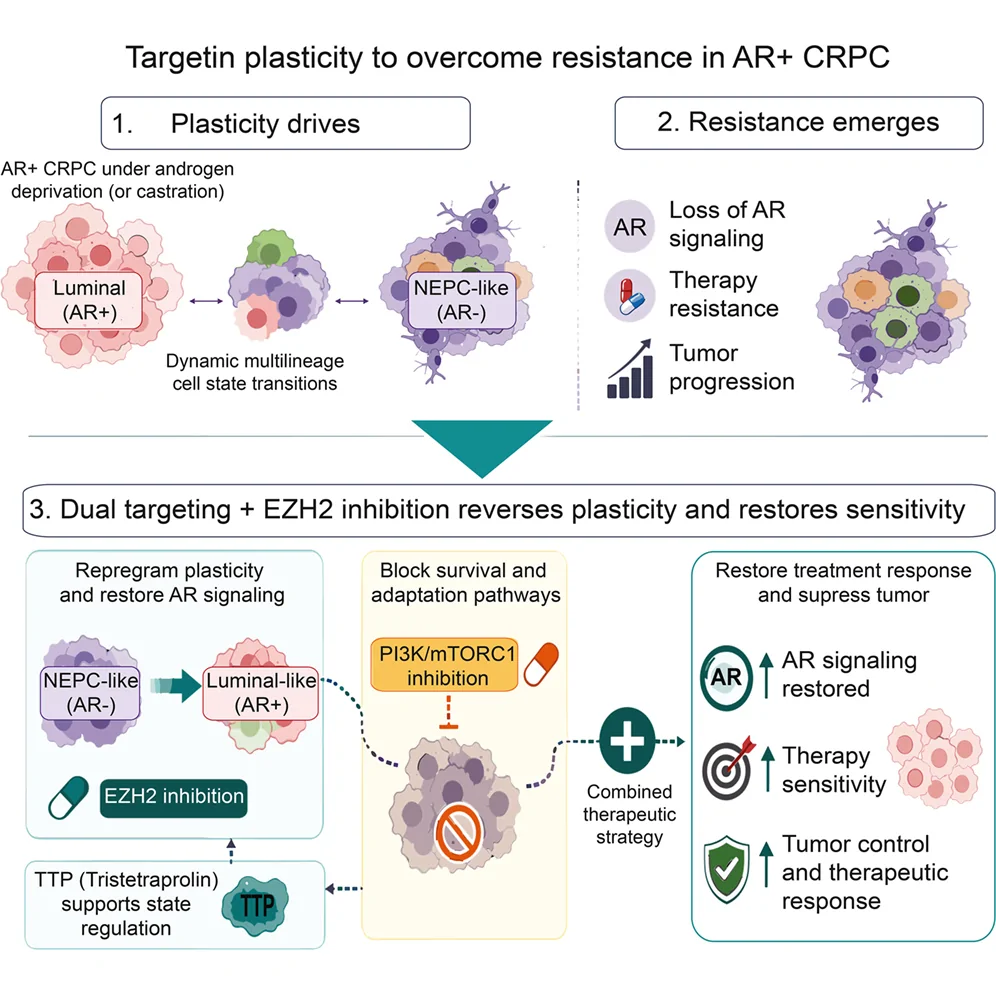

## Teaser

German et al. use integrated multi-omics analysis to identify EZH2 as a central regulator of prostate cancer phenotypic plasticity. The study reveals TTP-associated pathways and adaptive PI3K/mTORC1 as mediators of response to EZH2 inhibition and demonstrates that co-targeting EZH2 and PI3K/mTORC1 is an effective strategy in aggressive prostate cancer models.

## Introduction

Targeting androgen biosynthesis and androgen receptor (AR) function remains the cornerstone of treatment for prostate cancer (PCa) patients who have progressed beyond surgery and/or radiation therapies. While androgen receptor signaling inhibitors (ARSIs) offer a survival benefit, resistance is inevitable. Primary mechanisms driving resistance to ARSI involve the restoration of AR expression and function via AR amplification, mutation, or constitutively active AR splice variants, all of which sustain the AR-driven transcriptional program. Additional evidence demonstrates that approximately 15%–20% of patients develop ARSI resistance through a distinct mechanism termed phenotypic plasticity. This adaptive process involves epigenetic reprogramming and transcriptomic remodeling, often driven by loss-of-function alterations in key tumor suppressors such as PTEN, RB1, and TP53, which decouple tumor growth from AR dependency.^1^^,^^2^^,^^3^

These PCas often display altered kinase signaling and multilineage states involving activation of alternate transcriptomes.^4^ driving divergent cell identities including neuronal-, stem-, and basal-like gene signatures.^2^^,^^3^^,^^5^^,^^6^ These lineage-divergent states are marked by the emergence of alternate transcriptomes and lineage master regulators, contributing to therapeutic resistance and tumor aggressiveness. Importantly, phenotypic plasticity in PCa parallels similar processes observed in other solid tumors, including *EGFR*-mutant lung adenocarcinoma, Merkel cell carcinoma, and *BRAF* mutant melanoma, where transdifferentiation also underly therapeutic resistance.^7^^,^^8^^,^^9^ Although our insights into the molecular drivers of this plasticity are increasing, successful translation into effective therapies remains elusive. Research, including our own, has identified EZH2, the catalytic subunit of the polycomb repressive complex 2 (PRC2), as a key mediator that represses gene expression through histone H3 lysine 27 trimethylation (H3K27me3).^10^ In tumors exhibiting phenotypic plasticity, EZH2 is frequently overexpressed and silences differentiation-associated genes, thereby sustaining alternate lineage states. Loss of PRC2 in mouse models leads to the emergence of bivalent promoters marked by both active H3K4me3 and repressive H3K27me3 marks, features typically acquired during differentiation.^11^^,^^12^^,^^13^^,^^14^ Beyond its canonical methyltransferase activity, EZH2 also exerts non-catalytic functions, such as modulating mRNA translation and interacting with components of the translational machinery including restoration of AR expression, and response to enzalutamide.^2^^,^^3^^,^^15^

Aberrant activation of kinase signaling pathways has been reported in metastatic castration-resistant PCa (mCRPC) and is particularly pronounced in tumors undergoing phenotypic plasticity.^16^^,^^17^ Among these, the Janus kinase (JAK)-signal transducer and activation of transcription (STAT) signaling has emerged as a key driver, and inhibition of JAK-STAT signaling re-sensitizes phenotypic plastic PCa to enzalutamide by restoration of luminal lineage identity and AR signaling.^5^^,^^18^ These findings emphasize the importance of inflammatory and cytokine-driven signaling networks in facilitating lineage reprograming.

The PTEN/AKT/mTORC1 pathway, frequently activated through PTEN loss, is another critical axis in PCa, promoting cell survival, proliferation, and resistance to therapy. Canonically, EZH2 suppression inhibits mTORC1 signaling by derepressing its negative regulators such as TSC2, PTEN, and DEPTOR, reducing phosphorylation of 4E-BP1 and S6K, thereby inducing autophagy and apoptosis.^19^^,^^20^^,^^21^^,^^22^). EZH2 can also repress translation through direct interaction with eIF4E, independently of histone methylation.^23^ However, in specific oncogenic contexts such as NrasG12D-driven acute myeloid leukemia, EZH2 loss paradoxically activates mTORC1 through increased intracellular branched-chain amino acids.^24^ These context-specific findings underscore context-dependent effects of EZH2 on mTORC1 signaling, protein synthesis, and cancer progression.

Using a genetically engineered mouse model (GEMM) in which concurrent *Pten:Rb1* deficiency drives phenotypic plasticity characteristic of aggressive PCa, we demonstrate that EZH2 is a key regulator of multilineage states and their master regulator transcription factors (MR-TFs). Inhibition of EZH2 partially reverses these cell states and selectively modulates translational programs rather than global activation of protein synthesis. Consistent with this, ribosome profiling revealed gene-specific changes in translation efficiency that largely tracked with RNA abundance, without coordinated upregulation of ribosomal biogenesis or core translational machinery. Instead, EZH2 inhibition promotes targeted translational tuning of pathways involved in immune signaling, cellular plasticity, and microenvironmental interaction. These molecular shifts uncover context-specific vulnerabilities that can be therapeutically exploited. Co-inhibition of EZH2 and the PI3K/AKT/mTORC1 pathway produced superior antitumor activity in both murine and human preclinical models of PCa exhibiting phenotypic plasticity, particularly under castration where AR signaling is suppressed. Together, these data identify EZH2 as a key epigenetic gatekeeper of phenotypic plasticity and therapeutic resistance in PCa. Targeting EZH2 in combination with kinase signaling inhibitors may restore treatment sensitivity and suppress progression in aggressive PCa.

## Results

### EZH2 coordinates multi-lineage cell states mediated by RB1 loss

We previously demonstrated that combined loss of *Pten* and *Rb1* genes (DKO mice^3^) promotes a lineage transition from prostate adenocarcinoma toward a neuroendocrine-like (NE-like) or a highly plastic PCa phenotype that is resistant to ARSI. A central consequence of *Rb1* loss is the induction of EZH2 expression,^3^ the catalytic subunit of the PRC2 that drives widespread chromatin remodeling. Mechanistically, EZH2 establishes bivalent chromatin domains marked by the coexistence of H3K27me3, associated with transcriptional repression, and H3K4me3, associated with transcriptional activation.^11^^,^^13^ Such bivalent states are characteristic of developmentally poised genes and are increasingly recognized as a substrate for phenotypic plasticity in cancer. To explore EZH2-driven epigenetic reprogramming, we performed chromatin immunoprecipitation followed by sequencing (ChIP-seq) for H3K4me3 and H3K27me3 in DKO tumors and SKO tumors (*Pten* KO models, which retain an adenocarcinoma-like phenotype). Comparative analysis revealed chromatin redistribution of these histone marks in DKO tumors (Figures S1A–S1D). In particular, we observed repositioning and enrichment of H3K27me3 (Figure S1B), consistent with increased EZH2 activity and repression of lineage-specifying transcriptional programs. Importantly, pharmacologic inhibition of EZH2 partially reversed the NE-like phenotype, primarily through targeting bivalent chromatin-associated genes in DKO tumors (Figure S1E), and restored sensitivity to AR antagonism in this model,^3^ supporting a causal role for EZH2 in maintaining lineage infidelity.

To further define how EZH2 controls epithelial cell-state heterogeneity *in vivo*, DKO GEMMs were treated at 38 weeks of age with either vehicle control (*n* = 2) or the EZH2 inhibitor, EPZ011989 (*n* = 2) for 1 week. Tumors were subsequently subjected to single-nucleus RNA sequencing (snRNA-seq). A total of 10,000 *Epcam*^+^ (epithelial) nuclei were analyzed using an unbiased Leiden clustering approach and visualized by uniform manifold approximation and projection (UMAP) (Figure 1A). UMAP segregation based on treatment status revealed that EZH2 inhibition was responsible for 9 out of the 10 Leiden clusters identified (Figure 1A), indicating that EZH2 activity is a dominant regulator of transcriptional heterogeneity and multi-lineage cell state organization by controlling alternate transcriptomes. These results are in line with recent observations in both murine and human PCa devoid of *RB1* and/or *TP53* function.^5^

**Figure 1:**
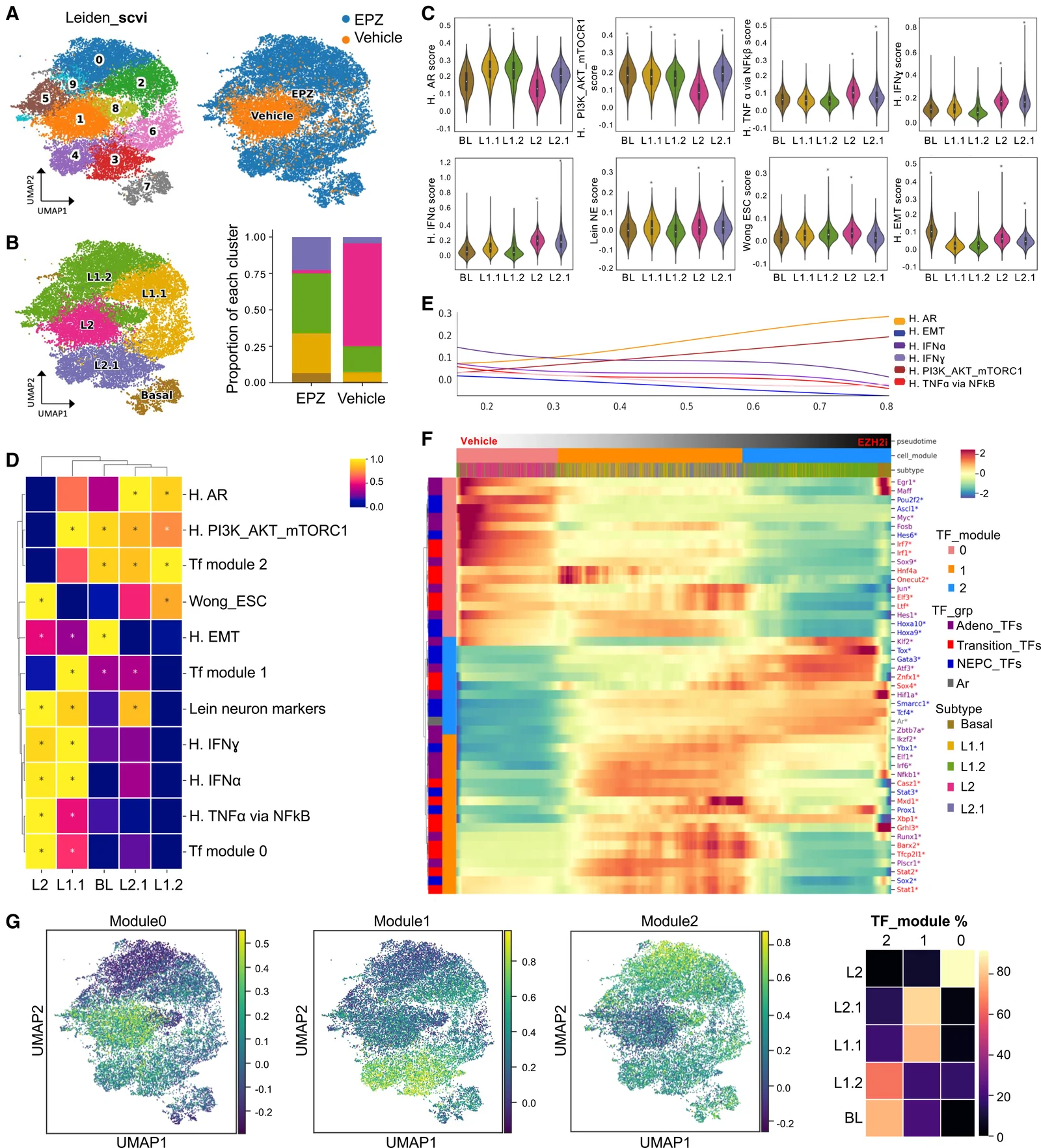
EZH2 coordinates multi-lineage cell states downstream of RB1 loss *PbCre:Pten:Rb1* KO (DKO) mice were treated daily for 7 days with vehicle (*n* = 2) or EZH2 inhibitor EPZ011989 (EZH2i, *n* = 2) via oral gavage at 38 weeks of age. Tumors were harvested, dissociated, and processed for single-nucleus RNA sequencing (snRNA-seq). (A) (Left) The uniform manifold approximation and projection (UMAP) visualization of Leiden-based unsupervised clustering of the epithelial cells (Epcam+). (Right) The UMAP showing treatment-specific distribution of epithelial cells from EZH2i-treated (blue) and vehicle-treated (orange) tumors. (B) UMAP and bars plot depicting epithelial subtype classification including basal-like (BL), luminal 2 (L2), luminal 2.1 (L2.1), luminal 1.1 (L1.1), and luminal 1.2 (L1.2) cells. (C and D) Violin plots and heatmaps showing significantly enriched gene sets across epithelial subtypes (BL, L1.1, L1.2, L2, and L2.2) in EZH2i-treated DKO tumors. Gene set enrichment analysis (GSEA) was performed with FDR <0.01 and absolute normalized enrichment score (NES) > 1 (see STAR Methods). H. AR: hallmark_AR (NELSON_ANDROGEN_SIGNALING_UP); H. PI3K_AKT_mTORC1: hallmark_ PI3K_AKT_mTORC1; H. IFNɣ: hallmark interferon gamma; H. TNF-α signaling via NF-κB: hallmark TNF-α signaling via NF-κB; H. IFN-α, hallmark of interferon alpha; Wong ESC, Wong embryonic stem cells; H. EMT, hallmark of epithelial mesenchymal transition. (E) Pseudotime analysis representing spline-fitted average *Z* scores of hallmark gene sets (hallmark of AR signaling, EMT, IFN-α, IFN-ɣ, PI3K-AKT-MTORC1, and TNF-α signaling via NF-κB) (see STAR Methods) from vehicle to EZH2i-treated tumors. (F) Heatmap of master regulatory transcription factors (MR_TFs) implicated in the transition from prostate adenocarcinoma to neuroendocrine prostate cancer (NEPC) across Epcam+ cells. Transcription factors groups (TF_grp) include adenocarcinoma-associated (adeno_TFs, purple), transition-associated (transition_TFs, red), and NEPC-associated (NEPC_TFs, blue) TFs. Cells are categorized into TF modules (modules 0–2) based on pseudotime, epithelial subtype, and MR-TF expression (scale imputed expression range: −0.5 to 1.5). (G) UMAP and heatmap visualization of the TF_grp expression across the TF_modules.

We next defined the sub-clusters using previously published gene-sets that define epithelial cell types during prostate development and PCa progression (Figure 1B; Figure S2A).^5^^,^^25^^,^^26^^,^^27^ Additional interrogation using gene set enrichment analysis (GSEA) enabled identification of both unique and shared biological programs across sub-clusters (Figures 1C and 1D; Figures S2B and S2C; Table S1). Vehicle-treated DKO tumors were dominated by a luminal-like cluster (L2) characterized by pronounced multi-lineage features, including inflammatory signaling, epithelial-mesenchymal transition (EMT), neuronal markers, and stemness-associated programs, coupled with strong repression of AR signaling and PI3K/AKT/mTORC1 activity (Figures 1C and 1D; Figures S2B–S2D). These transcriptional features were notably reversed following EZH2 inhibition, giving rise to multiple distinct luminal sub-clusters (L2.1 through L1.2) consistent with a stepwise reprogramming of epithelial cell states (Figures 1C and 1D; Figure S2D). In addition to luminal subpopulation, EZH2 inhibition induced the emergence of a basal-like epithelial cell population (Figures 1B–1D). While this cluster exhibited canonical basal markers (Figure S2A), it also exhibited strong enrichment for EMT and PI3K/AKT/mTORC1 signaling, along with moderate reactivation of AR-associated genes, suggesting a hybrid or transitional state (Figures S2A and S2D).

To further contextualize these cell states within established lineage trajectories, we leveraged a previously defined set of master regulator transcription factors (MR-TFs) derived from scRNA-seq analyses of phenotypic plasticity in PCa.^5^ These MR-TFs delineate transcriptional programs active in adenocarcinoma, transitional states, and established neuroendocrine prostate cancer (NEPC).^5^ Projection of these MR-TF modules into our snRNA-seq dataset revealed that NEPC-associated MR-TFs were enriched within the vehicle-treated L2 population, whereas EZH2 inhibition induced subpopulations enriched for transitional and adenocarcinoma-associated MR-TFs (Figures 1E and 1F). Visualization of these modules on the UMAP further highlighted the organization of lineage programs (Figure 1G). NEPC MR-TFs (module 0) localized primarily within L2 and L2.1 subpopulations; transitional MR-TFs (module 1) were enriched in L2.1, basal, and L1.1 clusters; and adenocarcinoma MR-TFs (module 2) predominated in basal, L1.1, and L1.2 clusters (Figures 1F and 1G). RNA velocity analysis, which infers cell trajectories and transitions across subclusters,^28^ indicates that EZH2 inhibition drives cell fate progression from the L2 (DKO vehicle-treated cells) toward the “most differentiated – L1.2” and to “least differentiated – L2.1” cell states, eventually leading to a basal-like cell population (Figure S2E). Pseudotime analysis confirmed this trajectory^29^ (Figure S2E). Collectively, these data underscore the central role of EZH2 in orchestrating epithelial lineage transitions downstream of RB1 loss. To investigate how EZH2 shapes the epigenetic landscape and contributes to phenotypic plasticity in PCa based on chromatin architecture, the ChIP-seq profiles of H3K4me3 and H3K27me3 were classified into four major states: (1) active (H3K4me3+ only), (2) repressed (H3K27me3+ only), (3) bivalent (both H3K4me3+ and H3K27me3+), and (4) unmarked (neither mark). This framework enabled systematic tracking of chromatin state transitions from SKO to DKO tumors and identification of chromatin configurations most affected by genotype (Table S2). Integration of ChIP-seq from SKO and DKO tumors with the snRNA-seq datasets from EZH2-inhibition-treated DKO tumors revealed that bivalent chromatin regions were preferentially remodeled (Figure S1F), suggesting a re-establishment of developmental poising. Representative examples of this remodeling included *Notch1*, *Tacstd2*, and *Mycn*, which were upregulated in DKO tumors following EZH2 inhibition (Figure S1E; Table S2). Notably, *Tacstd2* exhibited a shift toward a more repressive chromatin state in DKO tumors relative to SKO. However, upon EZH2 inhibition, *Tacstd2* expression was enriched in tumor cell clusters that adopt transcriptional features (L1.1 and L1.2) more like the SKO state. This indicates that its upregulation reflects a partial reversion of DKO cells toward an SKO-like lineage program, rather than a direct readout of the chromatin state observed in the untreated genotype comparison. Together, our data highlight a critical role for EZH2 in maintaining phenotypic plasticity in PCa models involving *Rb1* deletion and suggest that its inhibition may restore chromatin dynamics that constrain lineage infidelity.

### EZH2 regulates translational programs through canonical and non-canonical mechanisms to sustain phenotypic plasticity

Having established that EZH2 orchestrates epigenetic and transcriptional programs underlying phenotypic plasticity, we next sought to identify functional dependencies that could constitute therapeutic vulnerabilities. To this end, we performed an unbiased genome-wide CRISPR loss-of-function screen. DKO cells expressing Cas9 were transduced with the Brie lentiviral gRNA library, comprising 78,637 guides targeting 19,674 protein-coding genes.^30^ Cells were treated with either DMSO or the EZH2 inhibitor EPZ-6438, after which genomic DNA was isolated and single guide RNA (sgRNA) representation quantified by high-throughput sequencing (Figure 2A). Differential sgRNA abundance was assessed using the MAGeCK algorithm,^31^^,^^32^ revealing enrichment of genes involved in cell-cycle restraint and metabolic regulation in the EZH2-inhibited condition. Notably, top-ranked hits included components of E2F and DREAM-mediated cell-cycle repression (RBL1, E2F8, and LIN37), checkpoint signaling (Trp53), Hippo pathway components (NF2, AMBRA1), as well as multiple negative regulators of mTORC1 signaling (TSC1 and TSC2). In addition, genes encoding upstream inhibitors of nutrient-responsive mTORC1 activation were significantly enriched, including the GATOR1 complex (DEPDC5, NPRL2, and NPRL3), the Rag-lysosome regulator FLCN, endolysosomal trafficking proteins (VPS11 and VPS18), and PI3K pathway suppressors (INPPL1) (Figure 2A; Table S3). These findings suggest that cells under EZH2 inhibition become increasingly reliant on nutrient-sensing and growth signaling pathways. However, given the constitutive activation of PI3K/AKT signaling due to *Pten* loss in this model, these dependencies likely reflect context-specific vulnerabilities rather than *de novo* activation of anabolic translation programs.

**Figure 2:**
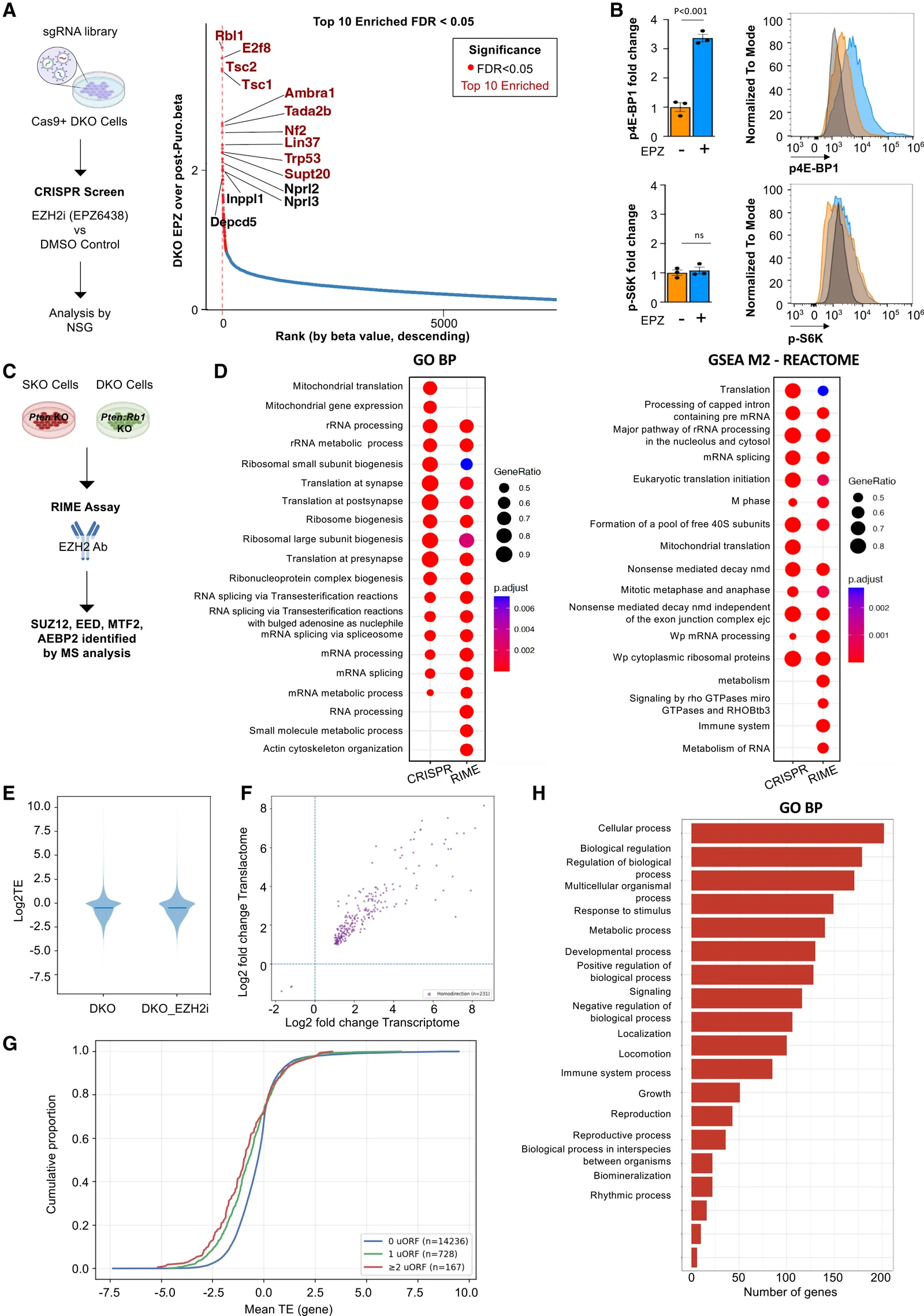
EZH2 regulates translation through canonical and non-canonical mechanisms to sustain phenotypic plasticity (A) Schematic overview of the CRISPR screen to identify EZH2 regulators. In brief, DKO cells were transduced with the Brie lentiviral gRNA library that consists of 78,637 gRNAs that target 19,674 protein coding genes and subjected to EZH2i (EPZ6438) treatment. Surviving/enriched populations were sequenced to identify genes whose loss conferred resistance or enhanced survival. (Right) Waterfall plot showing the top 10 positively selected genes from the genome-wide CRISPR knockout screen in DKO cells treated with vehicle or EZH2 inhibitor. Genes are ranked by β value (effect size), with positive values indicating enrichment under the indicated condition. Only significantly enriched hits are shown (FDR <0.05). (B) Bar graph and histogram showing fold changes in phosphorylated 4E-BP1 (p4E-BP1) and phosphorylated S6K (p-S6K) levels in DKO cells treated with EZH2 inhibitor (EPZ6438) versus DKO cells treated with DMSO. Data represent mean ± SEM from (*n* = 3) independent experiments. ns = no significant (two-tailed Student’s *t* test was performed). (C) Rapid immunoprecipitation mass spectrometry of endogenous proteins (RIME) was performed in SKO (*Pten* KO) and DKO (*Pten:Rb1* KO) cells using anti-EZH2 antibody to immunoprecipitated endogenous protein complexes from crosslinked chromatin. Mass spectrometry analysis identified associated proteins, revealing potential co-regulators and chromatin-bound interactors (*n* = 2). (D) Dot plot showing gene ontology biological process (GO-BP) and Reactome pathway enrichment across hits from the CRIPR screening and RIME analysis in SKO and DKO cells. Color indicates the *p* value, and dot size reflects the percentage of cells expressing the pathway-associated genes. (E) Violin plots showing the distribution of log2-transformed translational efficiency (log2TE) across the indicated experimental conditions. Each violin represents the kernel density estimation of log2TE values for individual genes within DKO cells with or without EZH2 inhibition. Central markers indicate the median and interquartile range (*n* = 3). (F) Scatterplot comparing gene-level changes in mRNA abundance and translational output within DKO cells with or without EZH2 inhibition. The *x* axis represents log2 fold change (log2FC) in transcript abundance (transcriptome), while the *y* axis represents log2 fold change (log2FC) in translational output (translatome). Each point corresponds to an individual gene. The diagonal indicates concordant regulation between transcription and translation, whereas deviations from this line highlight genes under post-transcriptional or translational regulation. (G) Cumulative distribution of genes stratified by upstream open reading frame (uORF) content (0, 1, or >2 uORFs) plotted against mean gene-level translation efficiency (TE). Genes were grouped based on the number of annotated uORFs in their 5′ untranslated regions, and mean TE was calculated per gene across samples. The cumulative proportion curves illustrate shifts in TE distribution associated with increasing uORF burden, highlighting a progressive reduction in translational efficiency with higher uORF content. (H) Bar plot showing Gene Ontology (GO) Biological Process (BP) enrichment analysis of genes exhibiting concordant (homodirectional) changes in both transcriptome and translatome in DKO cells treated with EZH2 inhibitor compared to control. Homodirectional genes were defined as those showing consistent up- or downregulation at both the mRNA and translational levels. Enriched GO terms were identified using over-representation analysis, and bars represent the significance of enrichment (−log10 adjusted *p* value) for each biological process. This analysis highlights coordinated transcriptional and translational programs affected by EZH2 inhibition.

To assess mTORC1 pathway activity, we quantified phosphorylation of downstream effectors by intracellular flow cytometry in DKO cells. EZH2 inhibition resulted in approximately 3-fold increase in phosphorylation of 4E-BP1 (p4E-BP1) relative to DMSO-treated controls (*p* < 0.001), consistent with enhanced cap-dependent translation initiation (Figure 2B). In contrast, phosphorylation of S6 kinase (p-S6K) remained unchanged (fold change around 1; *p* > 0.05) (Figure 2B). This pattern suggests that upstream pathway activation may not translate into coordinated increases in global protein synthesis.

To explore potential non-canonical mechanisms, we performed rapid immunoprecipitation mass spectrometry of endogenous peptides (RIME)^33^ to identify EZH2-interacting proteins in both SKO and DKO cells (Figure 2C). EZH2 protein pulldown robustly recovered canonical PRC2 components, including SUZ12, EED, MTF2, and AEBP2, confirming assay specificity (Figure 2C; Table S4). To biochemically validate EZH2 interactions and assess their dependence on nucleic acid scaffolding, we performed EZH2 immunoprecipitation followed by western blotting for PRC2 core and accessory subunits in SKO and DKO cells treated with EZH2 inhibitor, in the presence or absence of Benzonase. In SKO cells, EZH2 inhibition markedly reduced the association of EZH2 with the PRC2.1 subunit MTF2 and the PRC2.2 subunit EABP2, accompanied by diminished co-precipitation of the core components EED and SUZ12 (Figures S3A and S3B). Consistent with effective target engagement, flow cytometry analysis showed a reduction in H3K27me3 upon treatment (Figure S3C). EZH2 inhibition did not increase p4E-BP1 levels in SKO cells, indicating no activation of mTORC1-dependent translational signaling in this context (Figure S3C). These results indicate destabilization of both PRC2.1 and PRC2.2 complexes following EZH2 inhibition. In contrast, although H3K27me3 levels were similarly reduced upon treatment in DKO cells (FigureS3C), PRC2 interactions were largely preserved despite EZH2 inhibition (Figures S3A and S3B). Moreover, EZH2 inhibition led to a significant increase in p4E-BP1 levels (Figure S3C). This suggests that PRC2 complex stability is differentially dependent on EZH2 catalytic activity across genotypes. To identify high-confidence EZH2-interacting partners from the RIME assay, we applied a stringent false discovery rate (FDR) threshold of 0.1, requiring enrichment relative to the immunoglobulin G (IgG) control and detection in at least two out of the four samples. Functional enrichment analysis of EZH2-associated proteins revealed significant overrepresentation of pathways related to translation, RNA processing, and ribosome biogenesis (Figure 2D). While these interactions suggest a potential link between EZH2 and translational regulation, they do not establish direct functional control and may reflect broader chromatin-associated RNA regulatory complexes. Integration with the CRISPR screening and transcriptomic analyses revealed consistent enrichment of mTORC1-related signatures across datasets (Figure 2D; Figures S2B–S2D and S4A). To link these findings to cell-state dynamics, we examined gene signature scores derived from CRISPR and RIME analysis across the epithelial subclusters induced by EZH2 inhibition (L1.2, L1.1, L2.1, and BL) relative to the vehicle-treated (L2) cluster. Notably, ribosome biogenesis gene signatures were most enriched in the L1.2 ad L1.1 clusters, whereas translation-related pathways and capped intron pre-mRNA processing signatures were broadly distributed across all EZH2-inhibitor-induced subclusters (Figure S4B). This pattern closely paralleled a progressive increase in PI3K/AKT/mTORC1 signaling scores across these cell states. Together, these data indicate that EZH2 sustains phenotypic plasticity through integrated canonical chromatin-based repression and non-canonical regulation of translational machinery, enabling adaptive cell-stage transitions and survival in aggressive PCa.

To directly assess translational output, we performed ribosome profiling. Despite increased mTORC1 signaling, EZH2 inhibition did not induce a global shift in translation efficiency (TE) (Figure 2E). Instead, TE changes were largely concordant with RNA abundance, with most genes exhibiting homodirectional regulation between the transcriptome and translatome (Figure 2F). Consistent with this, Gene Ontology analysis revealed limited overlap between transcriptional and translational regulation at the pathway level (Figure S5A), indicating that TE changes are distributed across diverse genes rather than coordinated within canonical pathways. Analysis of upstream open reading frames (uORFs) confirmed their canonical repressive role in translation, as genes harboring multiple uORFs exhibited reduced TE in a dose-dependent manner (Figure 2G). However, this effect was not condition-specific, and uORF features, including abundance, GC content, and positional architecture, did not explain the translational differences observed following EZH2 inhibition.

Functionally, TE-associated pathways were enriched for broad biological processes including cellular signaling, cytoskeletal organization, and developmental programs, consistent with cell-state remodeling rather than increased anabolic output (Figure 2H). Notably, translationally upregulated genes included regulators of immune signaling and cell-state modulation (*Ptpn6*, *Gfi1*, *Slc7a11*, and *Cd34*) as well as mediator of cell-surface interaction and microenvironmental remodeling (*Ptprd*, *Sema3d*, *Abi3bp*, *Mme*, and *Fetub*). These genes were enriched for Gene Ontology terms related to *trans*-synaptic signaling and synapse organization (Figures S5B and S5C). Although classically associated with neuronal biology, these annotations capture broadly acting regulators of cell-cell communication, adhesion, and signaling interfaces, suggesting enhanced remodeling of microenvironmental interactions. Consistent with this observation, Gene Ontology molecular function analysis of translationally upregulated genes revealed significant enrichment for activities related to phospho-signaling regulation and transmembrane transport (Figure S5B). Notably, these enrichments were not observed in canonical immune or translational pathways, further supporting a model of selective, gene-specific translational tuning.

Collectively, these data support a unified model in which EZH2 sustains phenotypic plasticity through integrated canonical epigenetic regulation and non-canonical coupling to RNA and translational machinery. While upstream signaling pathways such as mTORC1 are engaged, this does not result in global activation of protein synthesis. Instead, EZH2 inhibition reveals a state of selective translational control that prioritizes specific gene programs involved in cell-state transitions and microenvironmental adaptation.

### Co-targeting EZH2 and PI3K/mTORC1 pathways as a treatment strategy for phenotypic-plastic prostate cancer

Collectively, our data demonstrated that pharmacologic inhibition of EZH2 consistently enriches for mTORC1-signaling-associated signatures, while functional genomics and proteomic analysis implicate EZH2 in the regulation of translational programs in models of phenotypic plasticity. Previously it has been demonstrated that aberrant translation in PCa cells is required to maintain AR-low PCa cells promoting tumor heterogeneity.^34^ Therefore, these observations led us to hypothesize that dual inhibition of EZH2 and mTORC1 signaling would more efficiently suppress adaptive survival mechanisms and thereby enhance anti-tumor efficacy. To test this hypothesis, we first evaluated therapeutic synergy in three-dimensional spheroid cultures derived from two genetically distinct models of phenotypic plasticity, including DKO (*Pten:Rb1* KO) and PPKO (*Pten:Trp53* KO). Spheroids were treated for three days with two independent EZH2 inhibitors, LY3346149 (LY-EZ) and EPZ6438 (EPZ), either alone or in combination with LY3023414 (LY-PK, a dual PI3K/mTORC1/2/DNA-PK inhibitor). Treatments were conducted under androgen-replete (FBS) or androgen-depleted castrate (CSS) conditions. Combination therapy produced the most pronounced synergistic anti-tumor effects under castrated conditions, whereas synergy was markedly attenuated in androgen-repleted settings (Figures 3A and 3B; Figure S6A). To directly assess downstream biological effects, we quantified cell death in organoid cultures following 72 h of treatment using immunofluorescence (propidium iodide and DAPI staining). Combination therapy markedly increased cell death relative to single-agent treatments, indicating that the observed synergy translates into enhanced cytotoxicity in phenotypically plastic tumor cells (Figure S6B). These results indicate that androgen deprivation unmasks a critical dependency on EZH2-mTORC1 crosstalk in phenotypically plastic tumor cells.

**Figure 3:**
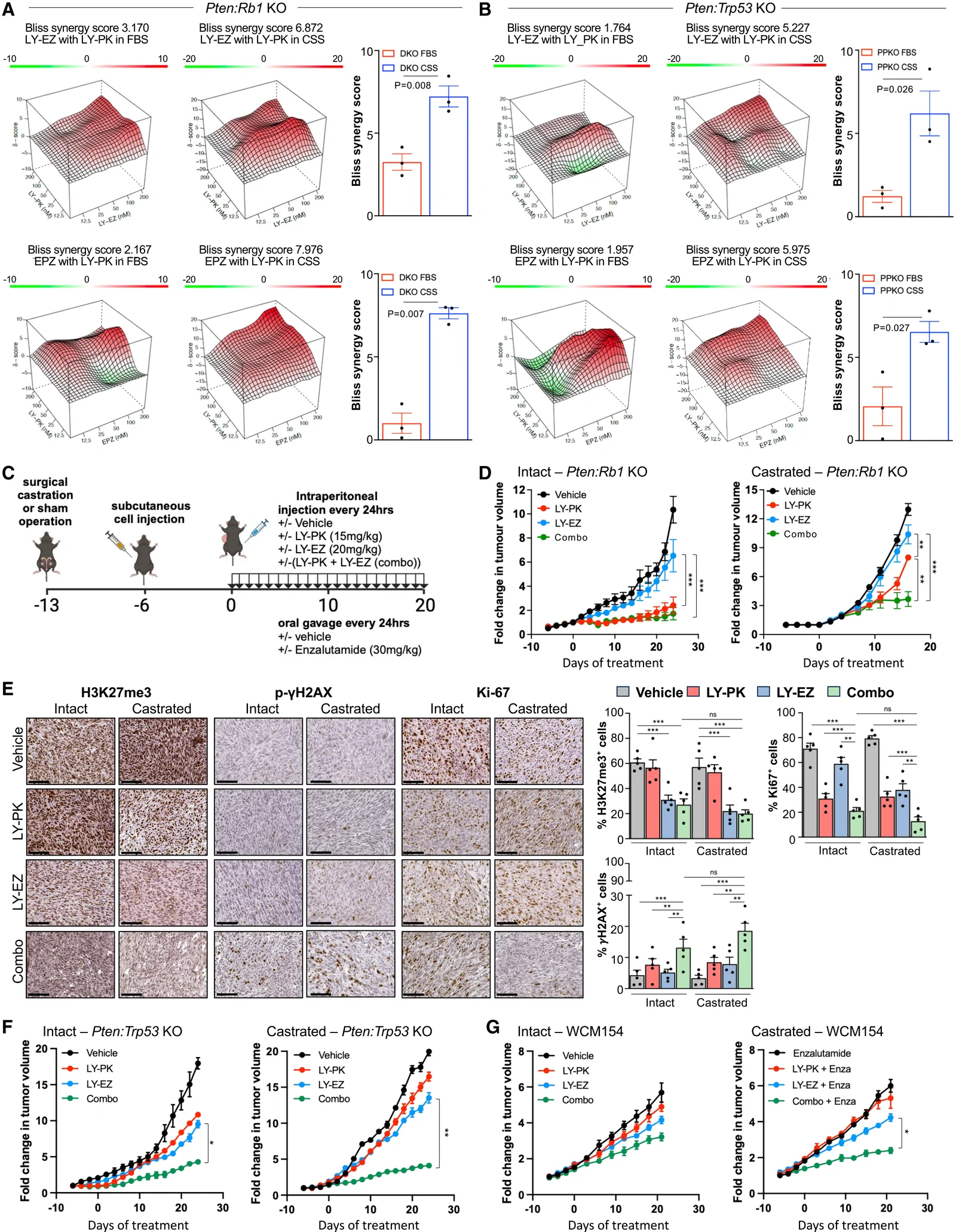
Combination of EZH2 and PI3K/mTORC1 inhibition as a therapeutic approach in phenotypic plastic prostate cancer (A and B) Bliss synergy landscapes generated using the Bioconductor package *synergyfinder* for drug combinations in 3D spheroid models. Graphs represent one representative replicate (with maximal synergy closest to its average value). Synergy score (*z* axis) ranges from −20 (green, antagonism) to +20 (red, strong synergy). *x* and *y* axes represent the concentrations of the two drugs (*n* = 3). (A) Drug combinations (LY-EZ with LY-PK [top] and EPZ with LY-PK [bottom]) tested in DKO spheroids under FBS and CSS (charcoal-stripped serum) conditions. (B) Same combination tested in *Pten:Trp53* KO (PPKO) spheroids. For specific drug concentrations, refer to Table S5. (C) Schematic representation of the experimental procedure to evaluate the therapeutic effect of combinatory therapies in three independent preclinical models. (D–F) Tumor growth curves showing fold change in tumor size over time under different treatment conditions (LY-PK, LY-EZ, and the combination). (D) Graphs representing the growth curves for DKO GEMM-derived allografts in C57BL/6N mice in intact or castrated conditions upon the specific treatments. In brief, C57BL/6N mice were surgical castrated, or sham operated at 8 weeks of age, and after 7 days castrated resistant 1 × 106 DKO cells were implanted into the flank of each mouse in 100 μL of Matrigel (50% in PBS). Once the tumors acquired 20 mm^2^, mice were treated daily with LY-PK (15 mg/kg) and/or LY-EZ (20 mg/kg) by intraperitoneal (IP) injection. During treatment, tumors were measured every second day for 20–30 days (*n* = 5 mice per treatment group [±SEM]). (E) H3K27me3, p-γH2AX, and Ki-67 immunohistochemistry staining in murine DKO (*Pten*^*:*^*Rb1* KO) tumors and corresponding staining quantification (*n* = 5 mice per treatment group, ±SEM). ∗∗= < 0.01, ∗∗∗= < 0.001, ns = not significant (two-tailed Student’s *t* test was performed). Scale bars = 50 μm. (F) Graphs representing the growth curves for PPKO (*Pten:Trp53* KO) GEMM-derived allografts in C57BL/6N mice in intact or castrated conditions upon specific treatments. (G) Graphs representing the tumor growth of 1 × 10^7^ human neuroendocrine PCa model (WCM154) in NOD.CB17-Prkdcscid/NCrCrl (NOD/LtSz) mice in intact or castrated conditions upon different treatments, following same schema than in (C). ∗*p* < 0.05, ∗∗*p* < 0.01 (two-tailed Student’s *t* test was performed).

We next extended these findings to *in vivo* models. DKO cells were subcutaneously injected into either intact or surgically castrated mice, which were subsequently treated with vehicle control, LY-EZ, LY-PK,^35^ or the combination of both agents (Figure 3C). LY-PK inhibition alone elicited a robust anti-tumor response in intact mice; however, this effect was diminished in castrated animals, indicating the emergence of acquired resistance under androgen-deprived conditions (Figure 3D). Importantly, the addition of EZH2 inhibition fully reversed this resistance in castrated mice, restoring tumor sensitivity to PI3K/mTORC1 blockade (Figure 3D). Combination therapy did not confer additional benefit over LY-PK monotherapy in intact tumors, suggesting that the therapeutic advantage of EZH2 inhibition is context-dependent and becomes most evident in castration-resistant disease. These findings implicate EZH2 catalytic activity as a key mediator of adaptive resistance to PI3K/mTORC1 inhibition in AR-dependent phenotypically plastic tumors. Histological analysis of formalin-fixed paraffin-embedded (FFPE) tumor sections confirmed effective target engagement, as evidenced by comparable loss of H3K27me3 following EZH2 inhibition across treatment groups. Further, combination therapy was associated with reduced tumor cell proliferation (Ki67), and increased double-strand DNA damage, as measured by p-γH2AX staining (Figure 3E). To assess the generalization of this therapeutic strategy, we validated these observations in two independent *in vivo* models, a murine model with combined deletion of PTEN and TP53 (PPKO),^36^ as well as a human NEPC organoid model^37^ transplanted *in vivo* (Figures 3F and 3G; Figure S7). In both systems, monotherapy with either EZH2 or PI3K/mTORC1 inhibitors produced only modest anti-tumor effects. In striking contrast, combined inhibition of EZH2 and PI3K/mTORC1, when administered alongside castration or enzalutamide, yielded the most significant and durable tumor suppression, significant loss of Ki67 staining, and increased double-strand DNA damage (Figure S7). These results closely mirror our findings in the DKO model, underscoring the robustness of this therapeutic vulnerability across genetically and biologically distinct models of PCa plasticity.

Drug tolerability was assessed in human prostate epithelial cells and wild-type animals (Figures S6C and S8). *In vitro*, treatment of primary prostate epithelial cells (PrECs) cultured under serum-free conditions with increasing doses of LY-PK in combination with either LY-EZ or EPZ did not result in reduced cell viability, as measured by CellTiter-Glo assays, nor did it increase cell death, as assessed by propidium iodide staining (Figure S6B). *In vivo*, tolerability was assessed by monitoring body weight, complete blood counts, and serum biochemical parameters, including markers of hepatic, renal, and electrolyte function. Given the known toxicity profiles of these agents, including hematologic effects associated with EZH2 inhibition^38^ and gastrointestinal toxicities linked to PI3K/mTORC1 pathway inhibition,^39^ we specifically evaluated parameters relevant to these liabilities. Combination treatment was well tolerated and did not result in additional toxicity beyond that observed with each agent alone. No significant changes in body weight, hematological parameters, serum chemistry, or organ function markers were detected across treatment groups (Figure S8). Importantly, no clinical signs of gastrointestinal toxicity, such as diarrhea or dehydration, were observed during the course of treatment.

Mechanistically, these observations are consistent with our previous work demonstrating that EZH2 inhibition restores AR expression and transcriptional activity in DKO cells,^3^ thereby re-sensitizing tumors to androgen deprivation and AR antagonism. Extending this observation, we found that LY-EZ similarly enhanced AR expression and activity in both DKO and PPKO cells (Figure S9), providing a mechanistic basis for the observed synergy between EZH2 inhibition, PI3K/mTORC1 blockade, and AR-directed therapies.

Collectively, these findings indicate that EZH2 promotes therapeutic resistance by coordinately suppressing AR signaling and sustaining adaptive translational programs. Simultaneous inhibition of these pathways represents a promising strategy to overcome castration-resistant PCa.

### Tristetraprolin contributes to EZH2-driven cell-state transitions and therapeutic efficacy

Our results demonstrate that EZH2 inhibition induces broad transcriptional changes associated with altered cell-state programs, including enrichment of translation-related signatures and reduction of inflammatory pathways mediated by interferon alpha (IFN-α), interferon gamma (IFN-γ), and tumor necrosis factor α(TNF-α)-nuclear factor κB (NF-κB) signaling. We previously demonstrated that deletion of TTP (*ZFP36*, gene encoding TTP) in a *Pten* KO GEMM causes widespread transcriptional alterations, predominantly affecting inflammatory and cell identity programs.^40^ We therefore investigated whether TTP-dependent programs contribute to the response to EZH2 inhibition and therapeutic efficacy.

Analysis of previously published mouse^3^ and human^2^ PCa gene expression datasets revealed that *ZFP36* expression is significantly reduced in phenotypic plastic/NEPC compared to adenocarcinoma samples (Figure 4A). Reduced TTP protein expression was also observed in our murine phenotypically plastic PCa models (Figure 4B). Following EZH2 inhibition, TTP protein levels increased in DKO cells (Figure 4B). Both snRNA-seq and bulk RNA-seq analyses showed only modest changes in *Zfp36* transcript expression following EZH2 inhibition (Figure S10A), suggesting post-transcriptional regulation. Consistent with this possibility, analysis of a TTP-associated gene signature derived from our *Pten:Zfp36* KO GEMM^40^ showed an overall downregulation following EZH2 inhibition, indicative of enhanced TTP functional activity (Figure 4C). TTP activity is also regulated post-translationally through phosphorylation.^41^ Accordingly, the catalytic subunit of protein phosphatase 2A (PPP2CA), which activates TTP through dephosphorylation, was enriched within epithelial subclusters induced by EZH2 inhibition (L1.2, L1.1, L2.1, and BL) (Figure 4D). These observations support the hypothesis that altered phosphatase-associated pathways may contribute to TTP regulation following EZH2 inhibition; however, whether EZH2 inhibition directly affects TTP phosphorylation status or activity requires further investigation.

**Figure 4:**
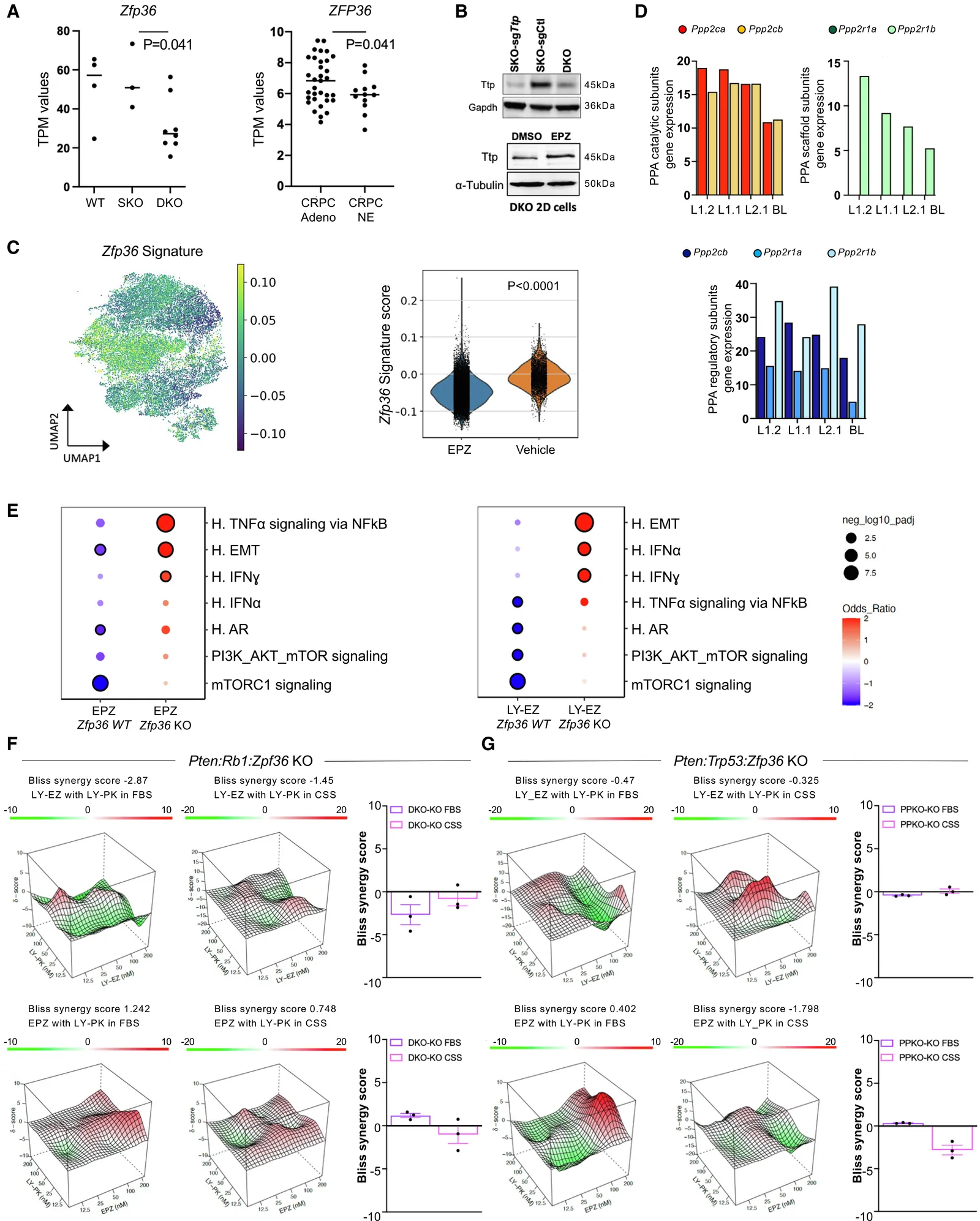
EZH2 inhibition cell state transition dependent on the RNA binding protein tristetraprolin (A) RNA-seq analysis of GEMM (wild-type [WT], SKO, and DKO) and human (castration-resistant prostate cancer [CRPC] and neuroendocrine prostate cancer [NEPC]) samples showing consistent downregulation of Zfp36/ZFP36 in NEPC. (B) Western blot showing Ttp protein levels in SKO Zfp36 KO, SKO control (Ctl), and DKO control cells (top) and DKO cells treated with DMSO or EPZ (5 μM) (bottom). (C) UMAP and violin plot of the *Zfp36* gene target signature generated from our *Pten:Zfp36* KO GEMM, demonstrating that EZH2 induces Ttp (Zfp36) function. (D) Bar plots showing the expression level of the genes encoding protein phosphatase 2A (PP2A) in L2 sub-cluster versus other sub-clusters (L2.1, L1.1, L1.2, and BL). (E) Dot plot of GSEA comparing gene signatures upregulated by EPZ or LY-EZ treatment in DKO *Zfp36* WT and KO cells. Color intensity represents average normalized enrichment score; dot size indicates the percentage of expressing cells. (E and F) Bliss synergy landscapes for LY-PK combined with LY-EZ or EPZ6438 in *Zfp36* KO spheroids. For specific drug concentrations, refer to Table S5 (*n* = 3). (F) Examples of the synergy profiles in DKO-*Zfp36* KO spheroids under FBS and CSS conditions. (G) Example of the synergy profiles in PPKO-*Zfp36* KO spheroids under the same conditions.

To assess the functional contribution of TTP in this context, we generated *ZFP36* knockout DKO and PPKO cell lines (Figure S10B). RNA-seq analysis following treatment with two independent EZH2 inhibitors (LY-EZ and EPZ-6438) demonstrated that loss of TTP altered the transcriptional response to EZH2 inhibition, including modulation of inflammatory gene programs and impaired induction of AR and mTORC1 signaling signatures (Figure 4E). Furthermore, combined EZH2 and PI3K/mTORC1 inhibition showed reduced efficacy in Zfp36-deficient DKO and PPKO models compared with parental cells (Figures 4E–4G; Table S5). These data indicate that TTP contributes to the cellular response to EZH2-targeted therapies and suggest that TTP-associated regulatory programs participate in maintaining the phenotypic state changes induced by EZH2 inhibition.

## Discussion

Phenotypic plasticity has emerged as a hallmark of cancer and a growing concern as a mechanism driving therapeutic resistance. Specifically, in PCa, phenotypic plasticity is thought to be a gateway for prostate adenocarcinoma to evolve by induction of a cell state switch toward a new molecular identity, including neuroendocrine PCa (NEPC).^3^^,^^5^^,^^42^^,^^43^^,^^44^ Previously, we observed that EZH2 inhibition partially reversed this lineage shift and restored sensitivity to ADT in part by restoration of AR expression and function.^3^ To more deeply address the regulation of cell identity changes governed by EZH2, we performed snRNA-seq using a PCa GEMM of phenotypic plasticity/NEPC driven by concurrent loss of *Pten* and *Rb1* (DKO). EZH2 inhibition in DKO mice significantly shifted cell identity. Using previously published PCa scRNA-seq datasets^5^^,^^34^^,^^45^^,^^46^ to annotate cell identity, we found that vehicle-treated DKO tumors predominately consist of L2/L2.1 progenitor-like cells (*Psca*^+^/*Krt4*^*+*^*/Tacstd2*^*+*^), with smaller subsets of L1/L1.1/L1.2 intermediate/differentiated luminal cell types, and luminal-basal cell populations. Following EZH2 inhibition, the L2 subpopulation was drastically decreased, while L2.1, L1-related subpopulations, and luminal-basal cells were increased. GSEA across subgroups further supported previous data^3^^,^^5^ where gene sets related to interferon and NF-κB inflammatory signaling, neuronal markers, and stemness were strongly enriched in DKO cells. Inhibition of EZH2 reduced these gene sets and increased AR signaling, which we previously reported,^3^ and PI3K-AKT-mTORC1 signaling was observed. Interestingly, while EMT genes were enriched in L2 subpopulations, they were most increased in the luminal-basal cell population specifically generated following EZH2 inhibition. These data coincide with previous publications demonstrating the importance of basal and basal-luminal cells for the initiation of PCa and phenotypic plasticity.^5^^,^^47^^,^^48^^,^^49^^,^^50^ In addition, MR-TFs driving transition of prostate adenocarcinoma to an NEPC phenotype were recently described.^5^ These MR-TFs were defined into three main modules representing adenocarcinoma (module 2), transition (module 1), and NEPC (module 0). These MR-TF modules were clearly represented with the NEPC module MR-TFs enriched in DKO-L2 (vehicle treated) and the transition and adenocarcinoma module MR-TFs enriched in DKO-L2.1 and DKO-L1 subgroups, respectively. Collectively, our data demonstrate the importance of EZH2 catalytic function to govern expression/action of MR-TFs that drive phenotypic plasticity and cell-state transitions, allowing prostate adenocarcinoma evolution to NEPC to evade AR signaling inhibitors.

While EZH2 is well established as a histone methyltransferase and transcriptional repressor within the PRC2 complex, accumulating evidence suggests that it also exerts non-canonical functions independent of its histone methyltransferase activity. These include roles in transcriptional activation,^51^^,^^52^ interaction with transcriptional machinery,^53^ and modulation of oncogenic signaling pathways such as AKT and Wnt.^54^^,^^55^ Building on this evolving understanding, our findings suggest that EZH2 may also influence translational regulation in a selective rather than global manner. Through integrated functional genomic and proteomic approaches, we identified a genetic dependence on translational-related processes in EZH2-driven contexts and observed that EZH2-associated protein complexes are enriched for factors involved in ribosome biogenesis and mRNA processing. Consistent with these findings, EZH2 inhibition resulted in selective activation of mTORC1 signaling, as evidenced by increased p4E-BP1 in the absence of corresponding changes in S6K phosphorylation, suggesting an uncoupled or partial activation state. However, ribosome profiling revealed that this signaling engagement does not translate into global increase in translational efficiency. Instead, TE changes were largely concordant with RNA abundance, with most genes exhibiting homodirectional regulation between the transcriptome and translatome, indicating that transcriptional outputs are largely propagated at the level of translation rather than independently reprogrammed. In line with this, functional enrichment of TE changes was limited and failed to identify coordinated activation of canonical translation machinery. This apparent discrepancy reflects a key distinction between gene-level concordance and pathway-level coordination. Although individual genes show consistent transcriptional and translational changes, these effects are broadly distributed across functionally diverse gene sets and therefore do not manifest as strong enrichment of specific biological pathways at the translational level. Integrating these findings with snRNA-seq trajectory analysis, which reveals EZH2-inhibition-driven transitions across distinct cellular states accompanied by increased PI3K/AKT/mTORC1 signaling, supports a model in which translational regulation is selectively tuned to reinforce cell-state transitions rather than globally enhanced. Together, these data suggest that EZH2 may contribute to phenotypic plasticity through coordinated transcriptional control and selective modulation of translation-related programs. This model is consistent with emerging evidence that translational control can fine-tune cell-state transitions in cancer without requiring increased overall protein synthesis.^49^^,^^53^

Motivated by these findings, we sought to identify rational combination therapies targeting the translational machinery, particularly in the context of NEPC, where plasticity-driven resistance remains a clinical challenge. Our previous data had demonstrated the efficacy of combining the HDAC inhibitor panobinostat and the dual PI3K/mTORC1 inhibitor BEZ235 in models of CRPC with PTEN deficiency.^56^ Similar results were observed using fimepinostat, a dual HDAC/PI3K inhibitor in NEPC preclinical models.^57^ Moreover, the dual PI3K/mTORC1 inhibitor gedatolisib has been shown to present greater anti-proliferative activity in PCa cells than single-target inhibitors,^58^ and combination strategies that include PI3K/AKT/mTORC1 and AR-targeting therapies have shown superior efficacy in androgen-sensitive PCa cell lines and PDX models.^59^ Using independent mouse models representing NEPC generated through co-deletion of *Pten and Rb1*^3^ or *Pten and Tp53*,^60^ along with a human NEPC model,^37^ we demonstrated that monotherapy with EZH2 or PI3K/mTORC1 inhibitor provided limited anti-tumor benefit in both intact and castrate settings. Modest responses to the combination therapy were seen in intact animals, but striking anti-tumor efficacy was observed under castrate conditions. Because tumor progression in these models involves a shift from AR-positive CRPC to AR-negative NEPC, we propose that EZH2 and PI3K/mTORC1 inhibitor co-treatment is particularly effective in castrate conditions due to EZH2 inhibition restoring AR expression and function. Additionally, we observed that EZH2 inhibition suppressed inflammatory signaling, consistent with prior work suggesting that inflammatory reprogramming is required for lineage transition from prostate adenocarcinoma to NEPC.^5^ Mechanistically, EZH2 has been shown to interact with RNA-binding proteins (RBPs) such as FBL (fibrillarin) or LIN28, influencing rRNA processing and the translation of specific oncogenic transcripts, including MYC and MYCN.^61^ These interactions enhance protein synthesis by promoting ribosome biogenesis or mRNA stability. We had recently shown that loss of the RNA-binding protein tristetraprolin (TTP), a key suppressor of inflammation, promotes tumor plasticity and resistance to enzalutamide in a mouse model of prostate adenocarcinoma.^40^ In this study, we found that CRISPR-mediated knockout of *Zfp36* altered the transcriptional response to EZH2 inhibition, including reduced suppression of inflammatory signaling and changes in AR and PI3K/AKT/mTORC1-associated pathways. Moreover, loss of TTP reduced the efficacy of combined EZH2 and PI3K/mTORC1 pathway inhibition in DKO and PPKO tumor cell models, particularly under castrate conditions. These findings suggest that TTP contributes to the cellular response to EZH2 and PI3K/mTORC1 co-inhibition and may participate in the regulation of cell-state transitions associated with therapeutic response.

In several cancer types, such as small cell lung cancer, it has been reported that alterations in chromatin remodeling result in transcriptional dysregulation, driving tumor cells toward a highly plastic state that leads to metastatic tumors.^62^^,^^63^^,^^64^ In PCa, it has been previously demonstrated that there are unique DNA methylation landscapes defining the transition from adenocarcinoma to NEPC.^2^ Further, it is shown that defined histone methylation (H3K4me3 and H3K27me3) landscapes emerged when the LNCaP adenocarcinoma cell line was engineered to overexpress MYCN, a known NEPC-related transcription factor.^65^^,^^66^ The association of retinoblastoma protein with chromatin is documented^67^ and demonstrates promiscuous activity, where it binds at promoters and enhancers in a cell-type-specific manner. In the absence of RB1, we observe a significant remodeling of H3K4me3 and H3K27me3 histone marks associated with promoter regions and activation of key genes implicated in NEPC, including Notch1,^68^ Tacstd2,^69^ and Mycn,^65^ highlighting RB1’s critical role in maintaining chromatin architecture. These findings align with previous reports showing that EZH2 inhibition can induce a poised chromatin state,^13^ further supporting its role in epigenetic regulation of plasticity.

Our findings demonstrate that EZH2 inhibition in phenotypic plasticity models driven by concurrent *Pten* and *Rb1* loss partially reverses NE-like states, restores AR signaling, and sensitizes tumors to AR-targeted therapies, particularly under castrate conditions. This reprogramming is accompanied by enrichment of PI3K/mTORC1 signaling and translation-related gene signatures, revealing a therapeutic vulnerability that can be exploited through combined EZH2 and PI3K/mTORC1 inhibition. Importantly, given the absence of global increases in TE, these effects likely reflect selective rewiring of signaling and gene expression programs rather than broad activation of protein synthesis. In contrast, Venkadakrishnan et al.^13^ report that EZH2 inhibition in fully established NEPC-patient-derived models does not restore luminal identity or AR expression; rather, it induces further upregulation of NE-lineage transcription factors such as ASCL1 and NEUROD1, reinforcing a terminally differentiated NE state. Their study highlights that bivalent promoters in NEPC are immediate targets of EZH2 inhibition and suggests that the non-canonical co-activator function of EZH2, which is prominent in PRAD and minimal in NEPC, may limit therapeutic efficacy. Consequently, they propose co-targeting cyclin/CDK2 as a rational strategy to overcome resistance. We have recently independently validated CDK2 as a viable therapeutic target using our phenotypic plastic models^64^ and agree this would be an exciting target to pursue. Together, these observations underscore a critical distinction between plasticity-driven NE-like states and fully committed NEPC. While EZH2 inhibition can reverse lineage infidelity in transitional contexts, it may consolidate NE identity in terminal states, necessitating biomarker-driven combination approaches tailored to the underlying differentiation status.

### Limitations of the study

First, although our findings were validated across complementary murine and human PCa models, the mechanistic studies were primarily performed in experimental systems that may not fully capture the complexity of advanced human disease. Second, while our data demonstrate that TTP contributes to the response to EZH2 inhibition and identify PI3K/mTORC1 inhibition as an effective combination strategy, additional studies will be required to establish the causal contribution of TTP and downstream translational programs to therapeutic response.

## Resource availability

### Lead contact

Further information and requests for resources and reagents should be directed to and will be fulfilled by the lead contact, Leigh Ellis (lellis@cpdr.org).

### Materials availability

This study generated DKO:Zfp36 KO and PPKO:Zfp36 KO cell lines. Requests for these materials should be directed to the lead contact. All materials will be made available upon completion of a material transfer agreement, where applicable.

### Data and code availability

• RNA-seq and single-cell RNA-seq datasets generated in this study have been deposited in GEO under accession number (Bulk RNA-seq [GEO: GSE306580], ChIP-seq [GEO: GSE306733], and snRNA-seq [GEO: GSE308826]). Ribo-seq data are provided in supplementary tables.
• Code used for snRNA-seq analysis is available at GitHub: https://github.com/KnottLab/PCa-EPZ-snRNA.
• Any additional information required to reanalyze the data reported in this paper is available from the lead contact upon request.

## Acknowledgments

We thank the Center for Functional Cancer Epigenetics at Dana-Farber Cancer Institute and the Center for Bioinformatics and Functional Genomics at Cedars-Sinai Medical Center for sequencing and data analysis support. D.P.L. is a William Dawson Scholar of McGill University, a Lewis Katz–Young Investigator of the 10.13039/100000892Prostate Cancer Foundation, and a Research Scholar–Senior from The Fonds de Recherche du Québec–Santé (https://doi.org/10.69777/380021). This study was supported by the Department of Defense Early Career Investigator Award (W81XWH-22-PRCP-EIRA to B.G. and W81XWH1910305 to K.L.M.), the 10.13039/501100000024Canadian Institutes of Health Research (PJT-203949 to D.P.L.), the NCI (R01CA252468 to L.E.), and the 10.13039/501100001809National Natural Science Foundation of China (82373106 to S.C.). The contents of this publication are the sole responsibility of the authors and do not necessarily reflect the views, opinions, or policies of 10.13039/100007188Uniformed Services University of the Health Sciences (USUHS), The Henry M. Jackson Foundation for the Advancement of Military Medicine, Inc., the 10.13039/100000005Department of Defense (DoD), or the Departments of the Army, Navy, or Air Force. Mention of trade names, commercial products, or organizations does not imply endorsement by the US Government.

## Author contributions

Conceptualization, L.E., B.G., and K.L.M.; methodology, L.E., B.G., K.L.M., T.N., N.B., F.D., X.Q., H.W.L., M.B., S.R.V.K., J.T.P., and D.P.L.; investigation, L.E., B.G., K.L.M., D.L.B., T.N., N.B., F.D., S.R.V.K., J.T.P., and D.P.L.; *in vivo* experimental design and animal ethics documentation, B.G. and D.L.B.; analysis, L.E., B.G., K.L.M., D.L.B., T.N., N.B., A.A.S., S.D., F.D., S.R.V.K., J.T.P., and D.P.L.; visualization, L.E., B.G., K.L.M., T.N., N.B., S.R.V.K., and D.P.L.; funding acquisition, L.E., B.G., K.L.M., and D.P.L.; project administration, L.E., B.G., and K.L.M.; supervision, L.E.; writing – original draft, L.E., B.G., K.L.M., D.L.B., T.N., N.B., F.D., M.L.F., S.R.V.K., J.T.P., and D.P.L.; writing – review and editing, L.E., B.G., K.L.M., D.L.B., T.N., N.B., F.D., M.L.F., S.R.V.K., J.T.P., and D.P.L.; and writing/editing, L.E. and B.G.

## Declaration of interests

The authors declare that they have no competing interests.

## STAR★Methods

### Key resources table

**Table:** 

REAGENT or RESOURCE	SOURCE	IDENTIFIER
**Antibodies**		

Tri-Methyl-Histone H3 (Lys4) Rabbit Monoclonal Antibody	Cell Signaling Technology	Cat#: 9751, C42D8; RRID:AB_2616028
Tri-Methyl-Histone H3 (Lys27) Rabbit Monoclonal Antibody	Cell Signaling Technology	Cat#: 9733, C36B11; RRID: AB_2616029
Rabbit monoclonal Ki67	Cell Signaling Technology	Cat#: 9129, D3B5; RRID:AB_2687446
Rabbit monoclonal phospho-H2AX	Cell Signaling Technology	Cat#: 9718, 20E3; RRID:AB_2118009
Rabbit monoclonal Anti-Androgen Receptor antibody [EPR1535(2)]	Abcam	Cat#:ab133273; RRID:AB_11156085
Rabbit monoclonal GAPDH	Cell Signaling Technology	Cat#: 2118, 14C10; RRID:AB_561053
EZH2 antibody (pAb) Rabbit polyclonal	Active Motif	Cat#: 39901; RRID: AB_2614956
Ezh2 Rabbit Monoclonal Antibody	Cell Signaling Technology	Cat#:5246, D2C9; RRIB:AB_10694683
SUZ12 Rabbit Monoclonal Antibody	Cell Signaling Technology	Cat#:3737, D39F6; RRID:AB_2196850
EED Rabbit Monoclonal Antibody	Cell Signaling Technology	Cat#:85322, E4L6E; RRIB:AB_2923355
MTF2 Rabbit Monoclonal Antibody	Cell Signaling Technology	Cat#:46454, F2X1A, RRID:AB_3755044
AEBP2 Rabbit Monoclonal Antibody	Cell Signaling Technology	Cat#:14129, D7C6X; RRID:AB_2798398
Polyclonal Rabbit anti-Human ZFP36/Tristetraprolin Antibody	LS Bio	Cat#: LS-B5606, aa41-90, RRID: AB_3755045
Tuberin/TSC2 Rabbit Monoclonal Antibody	Cell Signaling Technology	Cat#:4308, D93F12; RRID:AB_10547134
Phospho-S6 Ribosomal Protein (Ser235/236) Rabbit Monoclonal Antibody	Cell Signaling Technology	Cat#: 4858, D57.2.2E; RRID:AB_916156
Phospho-4E-BP1 (Thr37/46) Rabbit Monoclonal Antibody	Cell Signaling Technology	Cat#:2855, 236B4; RRID:AB_560835
Brilliant Violet 421™ Donkey anti-rabbit IgG (minimal x-reactivity) Antibody	Biolegend	Cat#: 406410; RRID:AB_10897810
Normal Rabbit IgG	Cell Signaling Technology	Cat#: 2729; RRID:AB_1031062

**Chemicals, peptides, and recombinant proteins**		

LY-PK, class I PI3K/mTOR/DNA-PK inhibitor	Lilly	Code name: LY3023414
LY-EZ, EZH2 inhibitor	Lilly	Code name: LY3346149
EPZ-6438 (Tazemetostat)	Selleck	Cat#: S7128, E7438

**Deposited data**		

Bulk RNA-seq	This paper	GEO: GSE306580
ChIP-seq	This paper	GEO: GSE306733
snRNA-seq	This paper	GEO: GSE308826
CRPC adenocarcinoma (CPRC-Ad) and CRPC-NE	Beltran H et al. (2016)^2^	dbGap phs000909.v.p1 and accessible on the cBIO Portal for Cancer Genomics^2^
Mouse bulk RNA-seq	Ku SY et al. (2017)^3^	GEO: GSE90891^3^

**Experimental models: Cell lines**		

Mouse Pten:Rb1 KO cell line	Dr. Leigh Ellis Laboratory	N/A
Mouse Pten:Trp53 KO cell line	Dr. Zongbing You laboratory	N/A
Human WCM154 organoids	Dr. Himisha Beltran	N/A
Mouse Pten:Rb1:Zfp36 KO cell line	Generated in this paper	N/A
Mouse Pten:Trp53:Zfp36 KO cell line	Generated in this paper	N/A

**Experimental models: Organisms/strains**		

Mouse: PBCre4:*Pten*^*f/f*^*: Rb1*^*f/f*^ in a mixed C57BL/6:129/Sv:FVB genetic background	Dr. Leigh Ellis laboratory	N/A
C57BL/6N mice	Charles River Labs	N/A
NOD.CB17-Prkdcscid/NCrCrl mice	Charles River Labs	N/A

**Recombinant DNA**		

lenti-CRISPR/Cas9v2 vector	Addgene	Cat#:5296
lentiviral Brie library (lenti-Guide-Puro backbone) system	J. G. Doench et al.^30^	N/A

**Software and algorithms**		

ImageJ	Schneider et al.	https://imagej.nih.gov/ij/
MAGeCK	W. Li et al.^32^	N/A
QuPath image analysis	NA	N/A
GraphPad Software	NA	N/A
Cell Ranger software	NA	N/A
Code	NA	https://github.com/KnottLab/PCa-EPZ-snRNA

### Experimental model and study participant details

#### *In vivo* therapy experiments

##### Mouse transplant models

Experiments were carried out using 8-week-old male C57BL/6N mice (Charles River Labs). Mice were subcutaneously injected with DKO or PPKO cells contained in matrigel/PBS (50/50). At time of tumor implantation, mice were either surgically castrated (castrated) or sham castrated (intact). Therapy started when tumors measured approximately 20mm^2^. Therapy consisted of daily of LY-PK (15mg/kg) and/or LY-EZ (20mg/kg) by intraperitoneal (IP) injection.

##### Mouse toxicity experiment

Experiments were carried out using 8-week-old male C57BL/6N mice (Charles River Labs). Mice were daily treated with LY-PK (15mg/kg) and/or LY-EZ (20mg/kg) by intraperitoneal (IP) injection during 10 days.

##### Human transplant model

Experiments were carried out using 8-week-old male NOD.CB17-Prkdcscid/NCrCrl mice (Charles River Labs). Mice were subcutaneously injected with WCM154 cells contained in matrigel/PBS (50/50). Mice were castrated 7 days before tumor transplant. Therapy started when tumors measured approximately 20mm^2^. Therapy consisted of daily of LY-PK (15mg/kg) and/or LY-EZ (20mg/kg) by intraperitoneal (IP) injection, with or without enzalutamide (30mg/kg) by oral gavage.

For all *in vivo* experiments, tumors were measured three times weekly by caliper measurements. Treatment toxicities were assessed by body weight, decreased food consumption, signs of dehydration, hunching, ruffled fur appearance, inactivity, or nonresponsive behavior.

All animal breeding and experiments were approved by and performed in accordance with the guidelines of the Cedars Sinai Medical Center, Institutional Animal Care and Use Committee (Animal protocol #9505).

#### Cell lines and organoid cultures

Murine *Pten:Rb1* knockout (DKO) 2D cell lines were generated from previously described genetically engineered mouse models.^3^ *Pten:Tp53* knockout (PPKO) 2D cell lines were kindly provided by Dr. Zongbing You.^36^ DKO and PPKO cell lines were cultured high-glucose DMEM with 1% P/S and either 10% FBS or CSS. Human WCM154 3D organoids have been described previously.^2^ Cell lines have been authenticated by genotyping PCR. Cell lines were tested for mycoplasma contamination.

### Method details

#### Drug response assays

For drug sensitivity assays, 500 or 350 cells/well for DKO and PPKO respectively were seeded in 3D in a 96 well plate pre-coated with collagen type I in suspension with 30% matrigel and treated with indicated concentrations of LY3023414 (LY-PK, class I PI3K/mTORC1/DNA-PK inhibitor), and LY3346149 (LY-EZ, EZH2 inhibitor), and EPZ6438 (EPZ, EZH2 inhibitor) for 72 h. Spheroid growth was assessed by CellTiter-Glo (Promega) assay according to manufactures instructions. Luminescence intensity of each well was normalized to the average of the control wells on the same plate to calculate relative cell viability values. For synergy analysis, relative cell viability measurements were analyzed using the web-based tool SynergyFinder.^70^ Bliss Synergy summary scores were derived from the average of the synergy scores across the entire dose–response landscape. Bliss analysis assume that the given drugs produce their anticancer effect by targeting different pathways, and that these pathways have no mechanistic connection other than the response outcome. Data visualization and statistical testing was performed using Prism 8 (GraphPad Software). Antibodies for ChIP-seq were H3K4me3 (C42D8) and H3K27me3 (C36B11) and purchased from Cell Signaling Technology. For immunohistochemistry, above histone antibodies were used, Ki67 (D3B5), and phospho-H2AX (20E3) antibodies were purchased from Cell Signaling Technology. For western blotting, androgen receptor antibody was purchased from Abcam and GAPDH from Cell Signaling Technology.

#### Single-nuclear RNA sequencing (snRNA-seq)

Nuclei were isolated from fresh frozen mouse prostatic tumors using a method modified from a recent single-nuclei RNA-sequencing (snRNAseq) study. The ST-SB buffer from that study was modified by removing Tween 20 and supplementing with 0.04 U/μL Protector RNase Inhibitor (Roche). All sample manipulation was performed on wet ice with wide-bore pipet tips (Rainin) and all centrifugations were performed with a swinging bucket rotor maintained at 4°C for 5 min at 850 × g. In brief, the frozen tissue was transferred onto a plate on dry ice and crushed into ≤1 mm3 pieces. This was then transferred to a 2 mL Dounce homogenizer (Kimble, cat: 885300-0002) on wet ice containing 1 mL of Nuclei EZ lysis buffer (Sigma, cat: NUC101). The tissue was then dunked approximately ×20 with Pestle A followed by ×20 with Pestle B. The lysis was then quenched by adding 1 mL of ST-SB. The sample was filtered through a pre-wetted 30 μm filter (Miltenyi Biotec, cat: 130–041-407) into a 15 mL conical tube. The homogenizer was rinsed 3× with 1 mL of ST-SB and this was transferred through the same 30 μm filter into the 15 mL conical tube. The sample was then centrifuged, the resulting supernatant removed, and the pellet resuspended with 500 μL of ST-SB. After, the sample was passed through a pre-wetted 20μm filter (Miltenyi Biotec, cat: 130–101-812) into a 1.5 mL protein lo-bind microcentrifuge tube (Eppendorf, cat: 022431081) and centrifuged. Nuclei concentration was quantified by mixing an aliquot of the sample with DAPI at a final concentration of 0.025 mg/mL in H2O. Samples were finally processed according to 10× Genomics protocol for the 3′ v3.1 assay and were super-loaded to target of 20,000 nuclei recovery. Single-cell RNA-Seq libraries were prepared per the 10× Genomics Single Cell 3′ v3.1 Reagent Kits User Guide using the Chromium Controller, X, or Connect (10× Genomics, Pleasanton, California). Barcoded sequencing libraries were quantified by quantitative PCR using the Collibri Library Quantification Kit (Thermo Fisher Scientific, Waltham, MA). Fragment analysis was performed on the 4200 TapeStation (Agilent Technologies, Santa Clara, California). Libraries were sequenced on a NovaSeq 6000 (Illumina, San Diego, CA) as per the Single Cell 3′ v3.1 Reagent Kits User Guide, with a sequencing depth of ∼40,000 reads/cell. The demultiplexed raw reads were aligned to the transcriptome using STAR (version 2.5.1)^71^ with default parameters, using mouse mm10 transcriptome reference from Ensembl version 84 annotation, containing all protein coding and long non-coding RNA genes. Expression counts for each gene in all samples were collapsed and normalized to unique molecular identifier (UMI) counts using Cell Ranger software version 4.0.0 (10× Genomics). The result is a large digital expression matrix with cell barcodes as rows and gene identities as columns.

#### Chromatin immunoprecipitation with sequencing (ChIP-seq)

Fresh-frozen prostatic tumor tissue was pulverized (Cryoprep Pulvrizer, Covaris), resuspended in PBS +1% formaldehyde, and incubated at room temperature for 20 min. Fixation was stopped by addition of 0.125 M glycine (final concentration) for 15 min at room temperature, then washing in ice-cold PBS + EDTA-free protease inhibitor cocktail (PIC; #04693132001, Roche). Chromatin was isolated from biological triplicates by the addition of lysis buffer (0.1% SDS, 1% Triton X-100, 10 mM Tris-HCl (pH 7.4), 1 mM EDTA (pH 8.0), 0.1% NaDOC, 0.13 M NaCl, 1× PIC) + sonication buffer (0.25% sarkosyl, 1 mM DTT) to the samples, which were maintained on ice for 30 min. Lysates were sonicated (E210 Focused-ultrasonicator, Covaris) and the DNA was sheared to an average length of ∼200–500 bp. Genomic DNA (input) was isolated by treating sheared chromatin samples with RNase (30 min at 37°C), proteinase K (30 min at 55°C) and de-crosslinking buffer (1% SDS, 100 mM NaHCO3 (final concentration), 6–16 h at 65°C), followed by purification (#28008, Qiagen). DNA was quantified on a NanoDrop spectrophotometer, using the Quant-iT High-Sensitivity dsDNA Assay Kit (#Q33120, Thermo Fisher Scientific). On ice, H3K27me3 (5 μL, #07–449, Millipore) or H3K4me3 (5 μL, #17–614, Millipore) antibodies were conjugated to a mix of washed Dynalbeads protein A and G (Thermo Fisher Scientific) and incubated on a rotator (overnight at 4°C) with 1.3 μg of chromatin. ChIP’ed complexes were washed, sequentially treated with RNase (30 min at 37°C), proteinase K (30 min at 55°C), de-crosslinking buffer (1% SDS, 100 mM NaHCO3 (final concentration), 6–16 h at 65°C), and purified (#28008, Qiagen). The concentration and size distribution of the immunoprecipitated DNA was measured using the Bioanalyzer High Sensitivity DNA kit (#5067-4626, Agilent). Dana-Farber Cancer Institute Molecular Biology Core Facilities prepared libraries from 2 ng of DNA, using the ThruPLEX DNA-seq kit (#R400427, Rubicon Genomics), according to the manufacturer’s protocol; finished libraries were quantified by the Qubit dsDNA High-Sensitivity Assay Kit (#32854, Thermo Fisher Scientific), by an Agilent TapeStation 2200 system using D1000 ScreenTape (# 5067–5582, Agilent), and by RT-qPCR using the KAPA library quantification kit (# KK4835, Kapa Biosystems), according to the manufacturers’ protocols; ChIP-seq libraries were uniquely indexed in equimolar ratios, and sequenced to a target depth of 40M reads on an Illumina NextSeq500 run, with single-end 75bp reads. BWA (version 0.6.1) was used to align the ChIP-seq datasets to build version NCB38/MM10 of the mouse genome. Alignments were performed using default parameters that preserved reads mapping uniquely to the genome without mismatches. Bam files were concatenated to sum the biological replicates of each state and bigwiggle files were calculated for comparing the different states.

#### Crispr screen

To carry out the pooled genome-wide Crispr screen, 1 × 10^8^ DKO-Cas9 cells were infected with the pooled lentiviral Brie library (lenti-Guide-Puro backbone) system^30^ at a multiplicity of infection of 0.5. Following 3 days of puromycin selection, half the cells were stored as day 0 control samples, and the remaining cells were cultured in DMSO or EPZ6438 (1μM) for an additional 2 weeks. PCR was carried using genomic DNA to construct the sequencing library which were then sequenced at approximately 30–35 million reads to achieve around 300× average coverage over the Crispr library. Data analysis was conducted using MAGeCK.^32^

#### RIME assay

##### Chromatin immunoprecipitation

Chromatin was isolated from fixed DKO and SKO cells by the addition of lysis buffer, followed by disruption with a Dounce homogenizer. Lysates were sonicated and the DNA sheared to an average length of 300–500 bp. Genomic DNA (Input) was prepared by treating aliquots of chromatin with RNase, proteinase K and heat for de-crosslinking, followed by ethanol precipitation. Pellets were resuspended and the resulting DNA was quantified on a NanoDrop spectrophotometer. Extrapolation to the original chromatin volume allowed quantitation of the total chromatin yield. An aliquot of chromatin (150 μg) was precleared with protein G agarose beads (Invitrogen). Proteins of interest were immunoprecipitated using 15 μl of antibody against EZH2 (Active Motif, Cat# 39901) and protein G magnetic beads. Protein complexes were washed then trypsin was used to remove the immunoprecipitate from beads and digested the protein sample. Protein digests were separated from the beads and purified using a C18 spin column (Harvard Apparatus). The peptides were vacuum dried using a speedvac.

##### Mass spectrometry

Digested peptides were analyzed by LC-MS/MS on a Thermo Scientific Q Exactive Orbitrap Mass spectrometer linked to Dionex Ultimate 3000 HPLC (Thermo Scientific) and a nanospray FlexTM ion source. The digested peptides were loaded directly onto the separation column Waters BEH C18, 75-micron x 100 mm, 130 Å 1.7u particle size. Peptides were eluted using a 100-min gradient with a flow rate of 323 nL/min. An MS survey scan was obtained for the m/z range 340–1600, MS/MS spectra were acquired using a top 15 method, where the top 15 ions in the MS spectra were subjected to HCD (High Energy Collisional Dissociation). An isolation mass window of 1.6 m/z was for the precursor ion selection, and normalized collision energy of 27% was used for fragmentation. A 20 s duration was used for the dynamic exclusion.

##### Database searching

Tandem mass spectra were extracted and analyzed by PEAKS Studio version 8 built 20. Charge state deconvolution and deisotoping were not performed. Database consisted of the Uniprot database (version 180508, 71,771 curated entries) and the cRAP database of common laboratory contaminants (www.thegpm.org/crap; 114 entries). Database was searched with a fragment ion mass tolerance of 0.02 Da and a parent ion tolerance of 10 PPM. Post-translational variable modifications consisted of methionine oxidation, asparagine and glutamine deamidation.

##### Criteria for protein identification

Peaks studio built-in decoy sequencing and FDR determination was used to validate MS/MS based peptide and the parsimony rules for protein identifications. A threshold of the −10∗logp (*p*-value) of 20 or greater was applied for the peptide identifications. The weighted sum of 9 parameters for peptide scoring are converted to a *p*-value which represent the probability of a false identification. Protein identifications were accepted if they could pass the −10logp of 20 and contained at least 1 identified unique peptide. Proteins that contained similar peptides and could not be differentiated based on MS/MS analysis alone were grouped to satisfy the principles of parsimony. Proteins sharing significant peptide evidence were grouped into protein groups.

##### List filtering

Protein coverage percentage is observed in the raw data file. Final list generation was done by taking all proteins with a spectral count of five and above from each replicate reaction and comparing them in a venn-diagram against IgG control replicates. Proteins unique to both experimental replicates were then applied to the PANTHER database for protein ontology results. Database *Searching*: Tandem mass spectra were extracted and analyzed by PEAKS Studio version 8 built 20. Charge state deconvolution and deisotoping were not performed. Database consisted of the Uniprot database (version 180508, 71,771 curated entries) and the cRAP database of common laboratory contaminants (www.thegpm.org/crap; 114 entries). Database was searched with a fragment ion mass tolerance of 0.02 Da and a parent ion tolerance of 10 PPM. Post-translational variable modifications consisted of methionine oxidation, asparagine and glutamine deamidation.

#### Ribosome profiling (Ribo-seq)

Ribosome profiling (Ribo-seq) was performed in DKO cells treated with vehicle or EZH2i to quantify genome-wide translation by isolating ribosome-protected mRNA fragments followed by deep sequencing. Ribosome footprints were mapped to coding sequences to determine ribosome occupancy, and translational efficiency (TE) was calculated by integrating Ribo-seq and RNA-seq data for each gene.

#### Co-immunoprecipitation

Co-immunoprecipitation was performed using the Universal Magnetic Co-IP Kit (Active Motif, #54002) following the manufacturer’s instructions. Briefly, whole-cell lysates were prepared in Complete Whole-cell Lysis Buffer supplemented with protease, phosphatase, and deacetylase inhibitors, clarified by centrifugation, and quantified by BCA assay. Lysates were treated with Benzonase in the presence of MgCl_2_ and quenched with EDTA. For immunoprecipitation, 500 μg of lysate was incubated with 5 μg of EZH2 antibody (Cell Signaling Technology, #5246) or rabbit IgG control (Cell Signaling Technology, #2729), followed by capture with Protein G magnetic beads. After washing, bound proteins were eluted in Laemmli sample buffer and analyzed by immunoblotting.

#### IHC staining and quantification of subcutaneously injected tumors

For all stainings, 5-μm thick sections were cut from paraffin-embedded blocks and dried onto positively charged microscope slides. The following primary antibodies were used: Ki67 (Thermo Fisher Scientific, D3B5, 1:100), phospho-H2AX (Cell Signaling Technology, 20E3, 1:100). And H3K27me3 (CellSignaling, C36B11, 1:400). Tissue sections were stained using the ImmPRESS HRP Anti-Rabbit IgG (Peroxidase) Polymer Detection Kit (Vector Laboratories). Tissue sections were imaged using a EVOS M7000 microscope scanner (ThermoFisher). Images were de-identified, and the full tissue sections were run through QuPath image analysis software to quantify positive DAB-stained cells (as a percentage of total cells).

#### Zfp36 CRISPR-knockout cell lines and RNA-seq

For CRISPR/Cas9-mediated knockout cell line generation in DKO and PPKO cells, guide RNA (gRNA) sequences CATGACCTGTCATCCGACCA and AAGCGGGCGTTGTCGCTACG targeting murine *Zfp36*, were cloned into the lenti-CRISPR/Cas9v2 vector (Addgene, #52961) according to the Zhang lab protocol. The scrambled gRNA sequence CACCGCGTGATGGTCTCGATTGAGT was used as a negative control. Viral infection was performed as described by the RNAi consortium (Broad Institute) laboratory protocol “Lentivirus production of shRNA or ORF-pLX clones,” and single clones were isolated following puromycin selection (2 μg/mL; Sigma-Aldrich #P8833). Cell lines have been authenticated by genotyping PCR and TTP protein staining by flow cytometry. Cell lines were tested for mycoplasma contamination. The DKO control (DKO Ctl) and DKO:Zfp36 KO (DKO KO) cells were treated with the indicated doses of LY3023414 (LY-PK, dual PI3K/mTORC1/2 inhibitor), LY3346149 (LY-EZ, EZH2 inhibitor), and EPZ6438 (EPZ, EZH2 inhibitor) for 96 h. RNA-seq data were aligned with STAR to mouse reference genome mm10 (GRCm38), quantified using RSEM and normalized. Gene set enrichment analysis (GSEA) was performed in GenePattern (genepattern.org). Genotypes were compared as described, using 10,000 gene set permutations to generate normalized enrichments scores, with FDR q-value <0.25 considered significant.

#### Western Blot

Sub-confluent treated cells were washed twice with cold PBS, trypsinized and then lysed in Pierce RIPA buffer (Thermo Fisher Scientific, 89900) with PhosSTOP inhibitor cocktail (Sigma Aldrich, PHOSS-RO) at 4°C for 30 min. Protein concentrations were measured by a Pierce BCA Protein Assay Kit (Thermo Fisher Scientific, 23225). Proteins were separated by SDS-PAGE (10% Mini-PROTEAN TGX Precast Gel, Biorad, 4561036) and transferred to a PVDF membrane (Bio-Rad, 1620177). The membrane was blocked 3% BSA in TBST for 1 h at room temperature and then blotted with primary antibodies overnight at 4°C. Blots were washed 3 × 5 min with TBST. The blots were then incubated with fluorescent-conjugated secondary antibody (Bio-Rad) 1 h at room temperature and washed 3 × 5 min with TBST. Proteins were visualized with a ChemiDoc MP fluorescent imager (Bio-Rad). Bio-Rad Image Lab analysis software was used to quantify protein band density. All western blots were repeated twice.

#### Intracellular antibody staining for flow cytometry

4–6 million cells were fixed 15 min with 4% PFA and permeabilized with 0.1% Triton by 30 min. After two washes with permeabilization buffer, cells were resuspended in FACS buffer containing antibody against Zfp36 (LS-B5606, LSBio), TSC2 (D93F12, Cell Signaling), p-S6K (9205, Cell Signaling), and p4E-BP1 (2855, Cell Signaling) and incubated 30 min at room temperature. After two washes cells were resuspended in FACS buffer containing the secondary antibody (Biolegend 406410). After two additional washes with permeabilization buffer, cells were resuspended in FACS buffer (1% of FCS+2mM EDTA in PBS).

### Quantification and statistical analysis

Statistical analyses were performed using GraphPad Prism and R, as indicated in the corresponding figure legends. Data are presented as mean ± SEM unless otherwise indicated. The number of independent biological replicates (n), statistical test used, and definition of error bars are provided in the corresponding figure legends. For comparisons between two groups, two-tailed Student’s t tests or appropriate non-parametric tests were used. For comparisons involving multiple groups or factors, one-way or two-way ANOVA with appropriate multiple-comparison correction was used. For high-throughput sequencing analyses, statistical significance was determined using the methods specified for each analysis, with correction for multiple testing where appropriate. Unless otherwise indicated, *p* < 0.05 was considered statistically significant. No data were excluded from analysis unless specified in the corresponding figure legend.

## Supplemental information

Document S1. Figures S1–S10

Table S1. snRNA-seq: EPZ vs. vehicle - clusters - GSEA

Table S2. Promoter_state_Histone-marks_DEG

Table S3. Crispr screen gene summary

Table S4. Differential from RIME assay

Table S5. Bliss synergy landscapes for LY-PK and LY-EZ or EPZ6438 drug combinations

Document S2. Article plus supplemental information
