# Supplementary material for: EZH2-dependent phenotypic plasticity reveals TTP-associated and mTORC1 therapeutic vulnerabilities in lethal prostate cancer
Source: Cell Rep Med. 2026 Sep 30;7(10):103083. doi: 10.1016/j.xcrm.2026.103083 (PMC13710546; doi:10.1016/j.xcrm.2026.103083)
Supplement: Supplementary file 1 [file mmc1.pdf]

Cell Reports Medicine, Volume *7*

Supplemental information

EZH2-dependent phenotypic plasticity reveals TTP-associated and mTORC1 therapeutic vulnerabilities in lethal prostate cancer

Beatriz German, Katherine L. Morel, Teia Noel, Nadia Boufaied, Deborah L. Burkhart, Sujun Chen, Felipe Dezem, Xintao Qiu, Henry W. Long, Kun-Lin Ho, Stefan DiFazio, Sylvan Baca, Ayesha A. Shafi, Matthew L. Freedman, Himisha Beltran, Christopher J. Sweeney, Housheng Hansen He, Myles Brown, Jasmine T. Plummer, Simon.R.V. Knott, David P. Labbé, and Leigh Ellis

Supplementary Figure 1: RB1 loss remodels chromatin states across transitional cell programs

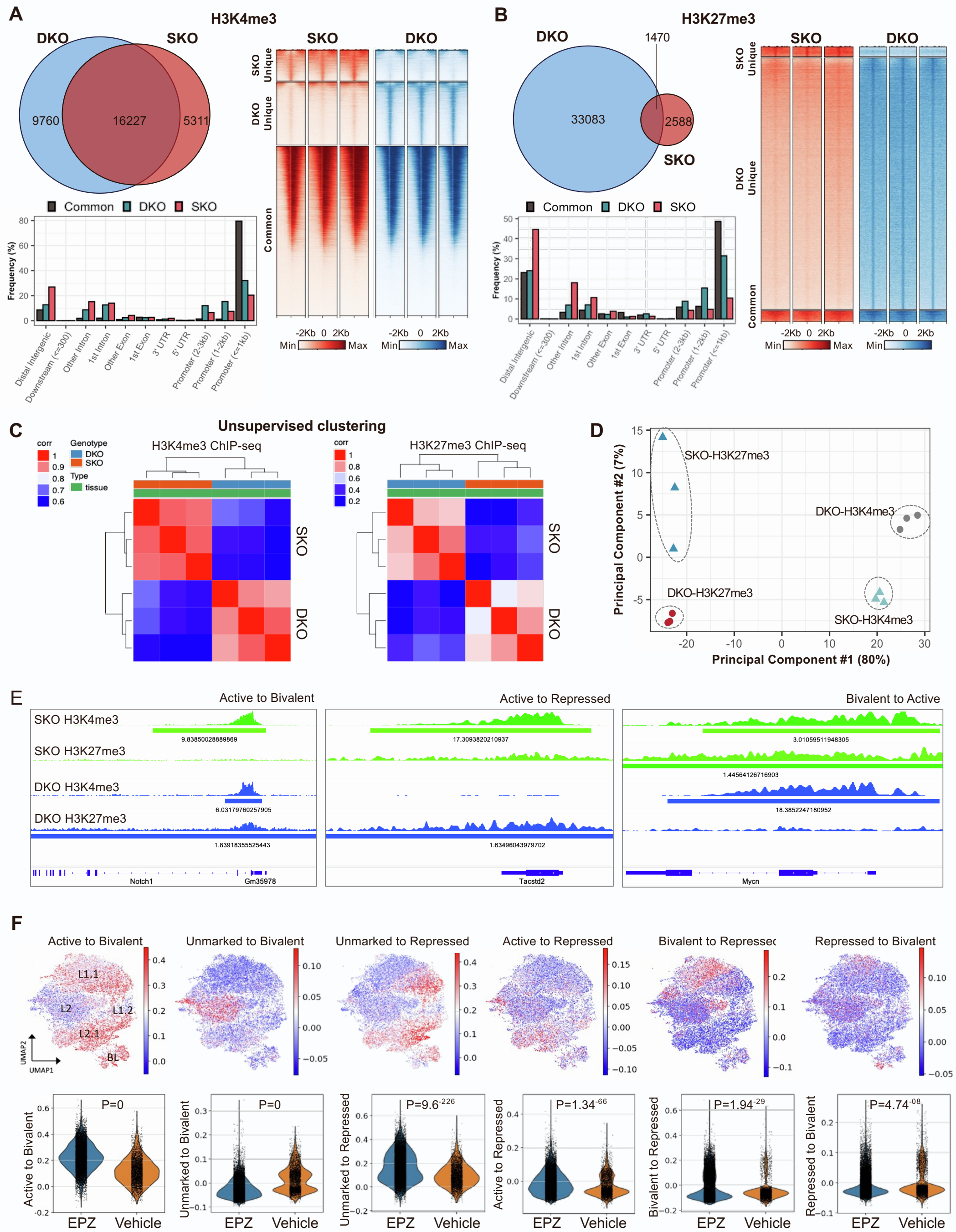

Supplementary figure 1:

EZH2 coordinates multi-lineage transitional cell states mediated by RB1 loss. **A**. Venn diagram (left) and tornado plot (right) illustrating the 31,298 ChIP-seq peaks for H3K4me3 in *PbCre:Pten KO* (SKO) and *PbCre:Pten:Rb1 KO* (DKO) tumors. Bar plot (bottom left) indicates H3K4me3 peak genome-wide peak distribution. 52% of the peaks were shared between SKO and DKO, 17% and 31% were considered unique in SKO and DKO respectively. The analysis of exact genomic location of H3K4me3 is illustrated in the bar plot and showed that most of the genomic locations are at promoter regions. **B.** Venn diagram (left) and tornado plot (right) illustrating the 37,141 ChIP-seq peaks for H3K27me3 in SKO and DKO tumors. 4% of the peaks were shared between SKO and DKO, 7% and 89% were considered unique in SKO and DKO respectively. The analysis of exact genomic location of H3K27me3 is illustrated in the bar plot and showed that most of the genomic locations are at promoter regions. **C.** Hierarchical clustering of SKO and DKO tumors based on sample-to-sample correlation of H3K4me3 and H3K27me3 peaks representing promoter occupancy. **D.** Two-dimensional principal component analysis (PCA) of H3K4me3 and H3K27me3 peaks representing promoter occupancy **E.** Gene track examples chromatin remodeling in Notch1 and Tacstd2 which move from an active to bivalent or repressed state respectively and Mycn from a bivalent to active state. **F.** UMAP representing the genes transitioning from Active to Bivalent, Unmarked to Bivalent, unmarked to repressed, active to repressed, bivalent to repressed and repressed to bivalent from SKO to DKO tumors based on the H3K4me3 and H3K27me3 ChIP-seq analysis (top). Violin plots of significantly enriched gene sets in DKO upon vehicle or EPZ treatment. FDR < 0.01, abs (NES) > 1 where NES is the normalized enrichment score.

Supplementary Figure 2: EZH2 inhibition reshapes epithelial cell states

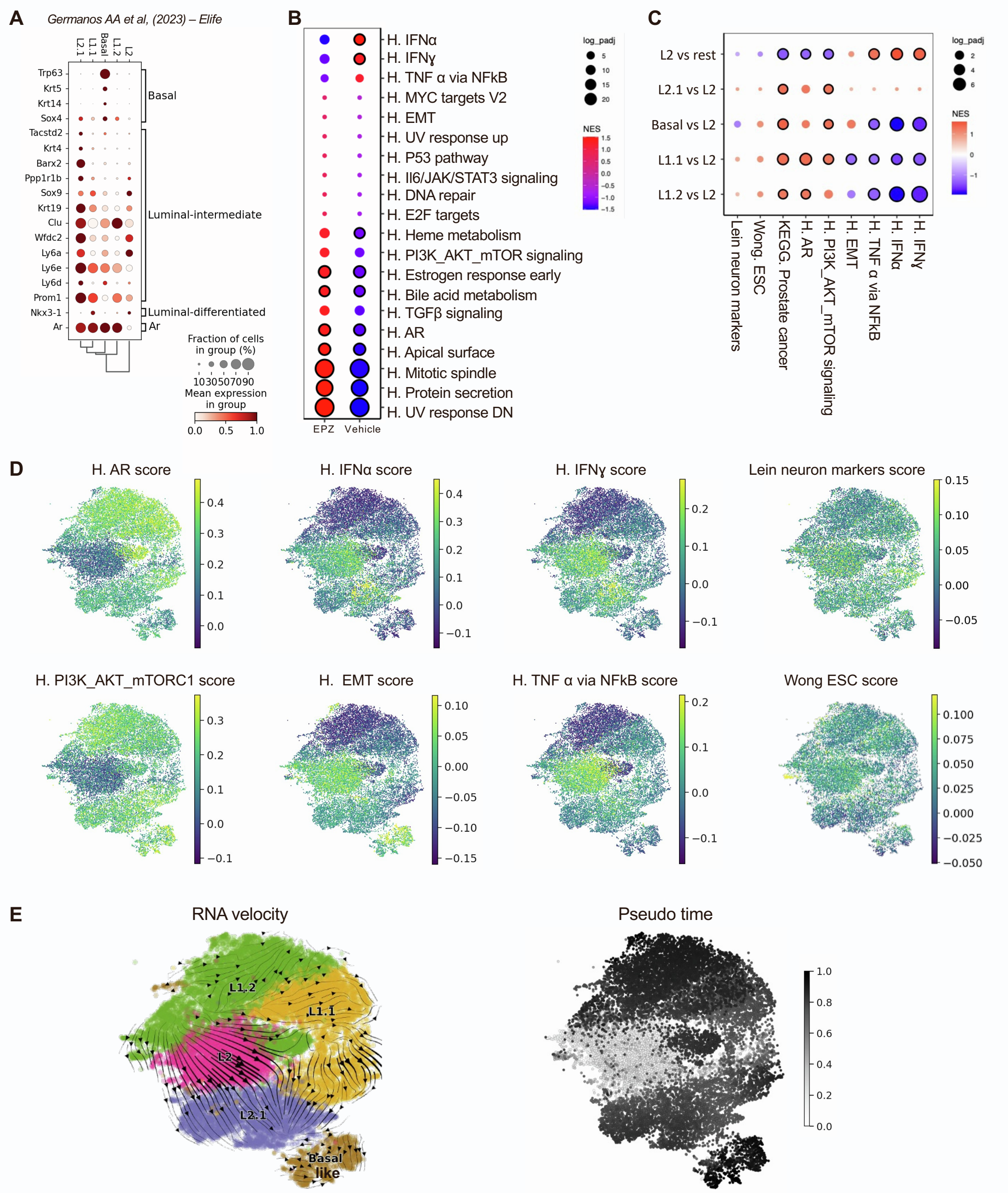

Supplementary Figure 2:
A. Dot plot of canonical marker genes for each cell type. Hue of red indicates average normalized gene expression level and size of dot indicates percentage of nonzero gene expression for each cell population. **B.** Dot plot of the Gene Set Enrichment Analysis (GSEA) of transcripts upregulated in EPZ treated mice compared with vehicle. **C.** Dot plot of the GSEA of transcripts upregulated in each epithelial sub-cluster compared with L2. **D.** UMAP of the androgen signaling (NELSON_ANDROGEN_ SIGNALING_UP), epithelial mesenchymal transition (EMT), interferon alpha response (IFNα), interferon gamma response (IFNɣ), PI3K-AKT-MTOR signaling and TNFα signaling via NFkB, embryonic stem cells. **E.** RNA velocity analysis and pseudo time analysis of epithelial cells in vehicle and EPZ treated mice.

Supplementary Figure 3: EZH2 interactome is context dependent

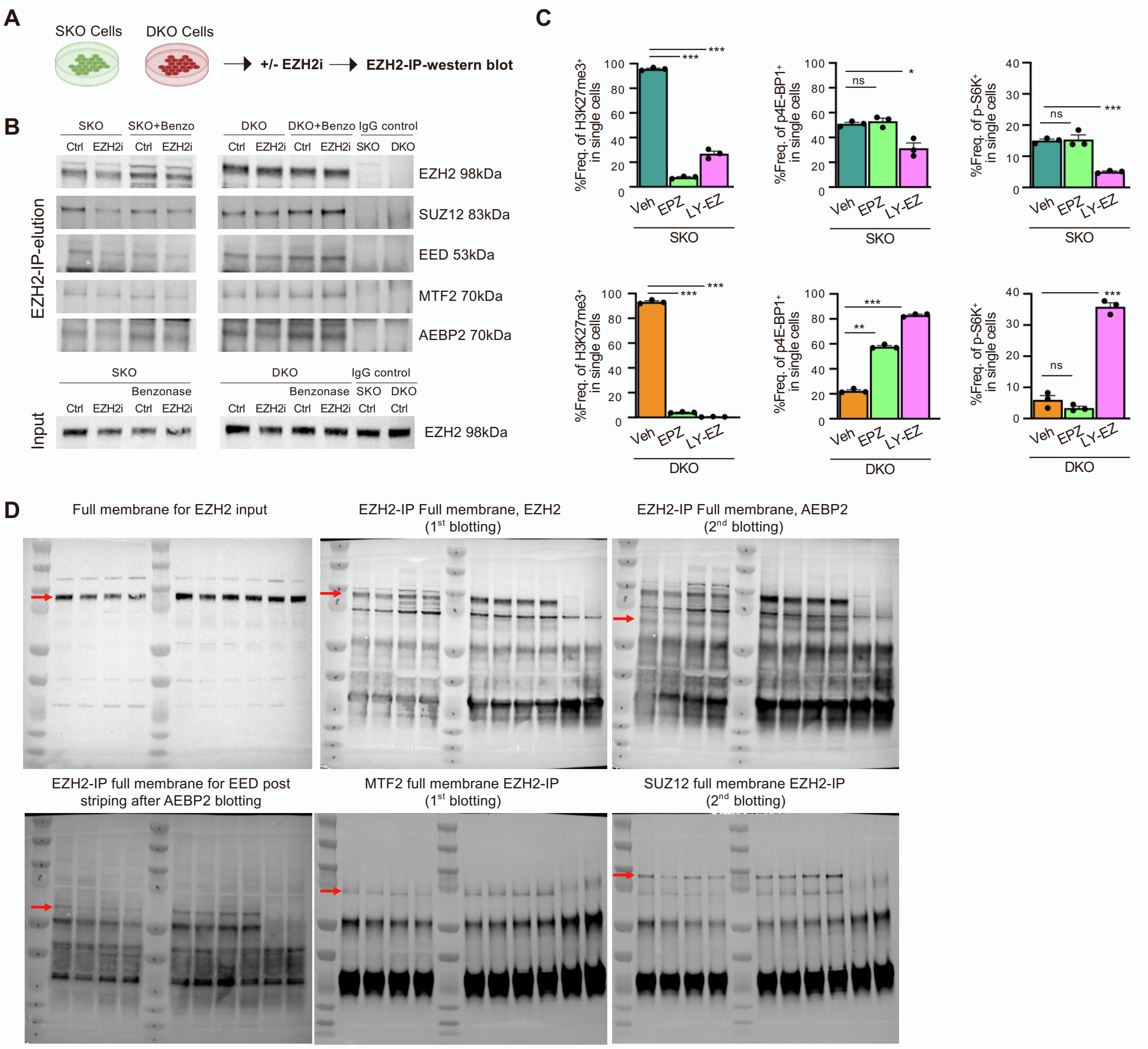

Supplementary figure 3: A. Schematic overview of the experimental plan. In brief SKO and DKO cells were treated with EZH2 inhibitor following by Benzonase treatment and EZH2-IP analyzed by western blot (WB). **B.** WB analysis of EZH2-IP including PRC2 components SUZ12, and EED, the PRC2.1 subunit MTF2 and PRC2.2 subunit AEBP2 in SKO and DKO cells treated with EZH2 inhibitor (EPZ, 5μM, 96hrs). **C.** Bar plots representing flow cytometry analysis of H3K27me3, p4E-BP1, and p-S6K in SKO and DKO cells treated with vehicle, EZH2 inhibition (EPZ and LY-EZ) at 200nM, 72hrs. Data represent mean ± SEM from [n=3] independent experiments. *= <0.05, ** = <0.01, *** = <0.001, ns= no significant. **D.** Full membrane blots for EZH2 immunoprecipitation (EZH2-IP).

Supplementary Figure 4: CRISPR and RIME analyses identify translation-related programs associated with epithelial cell states

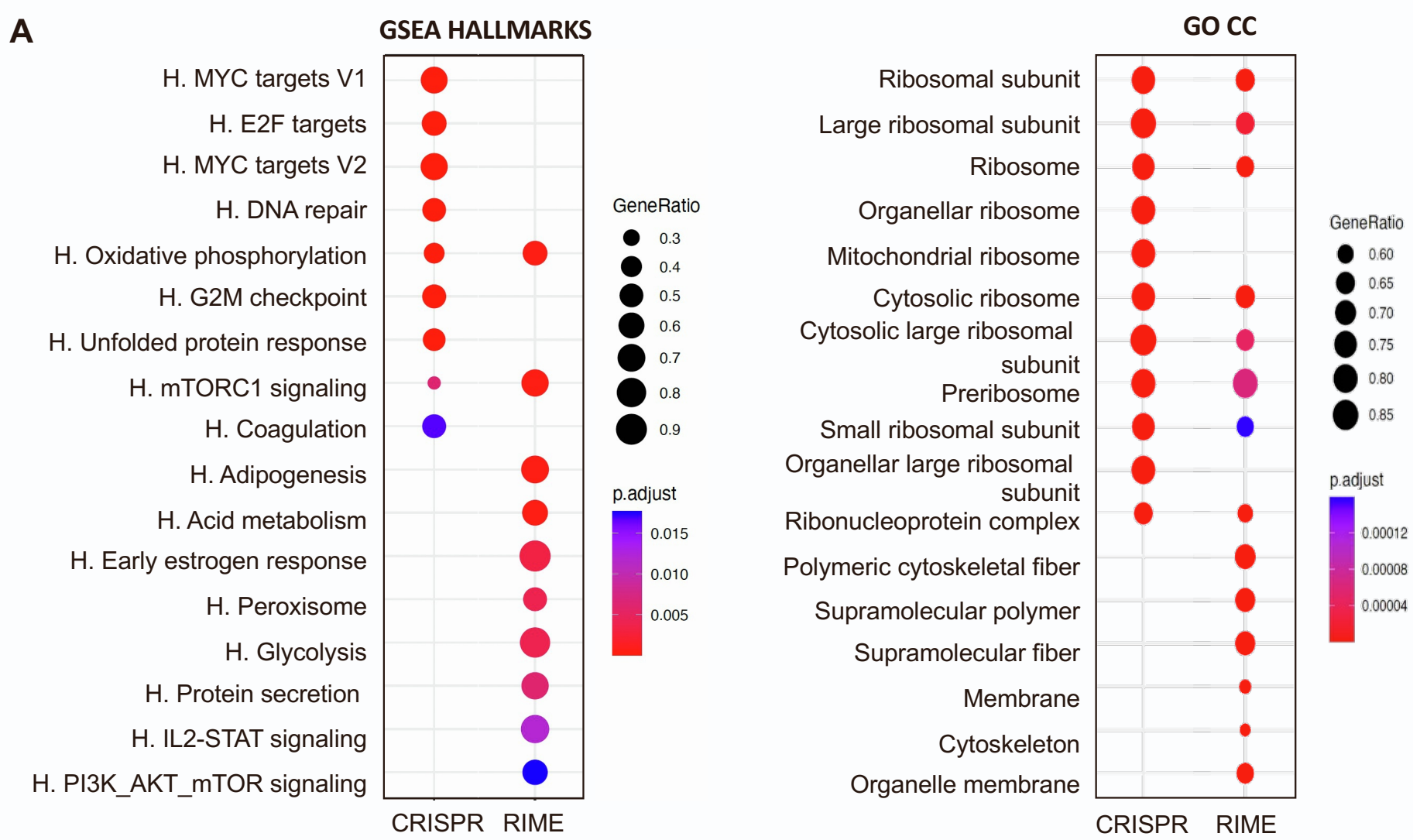

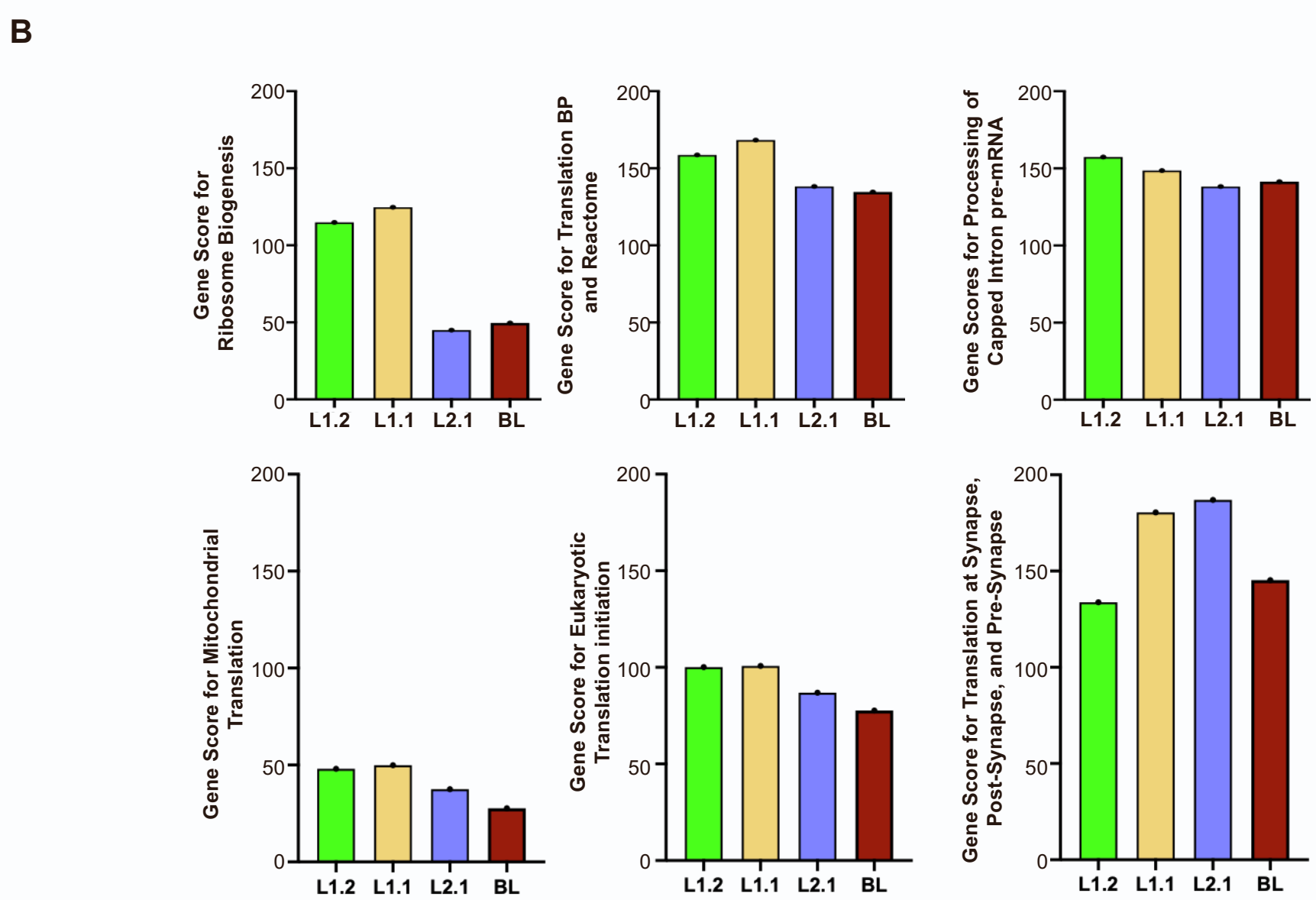

Supplementary Figure 4:
A. Dot plot representing the Gene Set Enrichment Analysis (GSEA) Hallmarks pathway and gene ontology cellular component analysis demonstrate overlay of common pathways and process's from Crispr screening and RIME analysis for DKO and SKO cells. The blue and red color represents the p-values across all cells, and the size of the dot corresponds to the percentage of cells expressing the characteristic for each pathway. **B.** Bar plots showing the signature related with translation mechanisms in L2 sub-cluster versus other sub-clusters (L2.1, L1.1, L1.2 and BL). The analyzed signatures include genes related with processing of capped intron pre-mRNA, eukaryotic translation initiation, and mitochondrial translation.

Supplementary Figure 5: EZH2 inhibition alters both transcriptional and translational programs

A

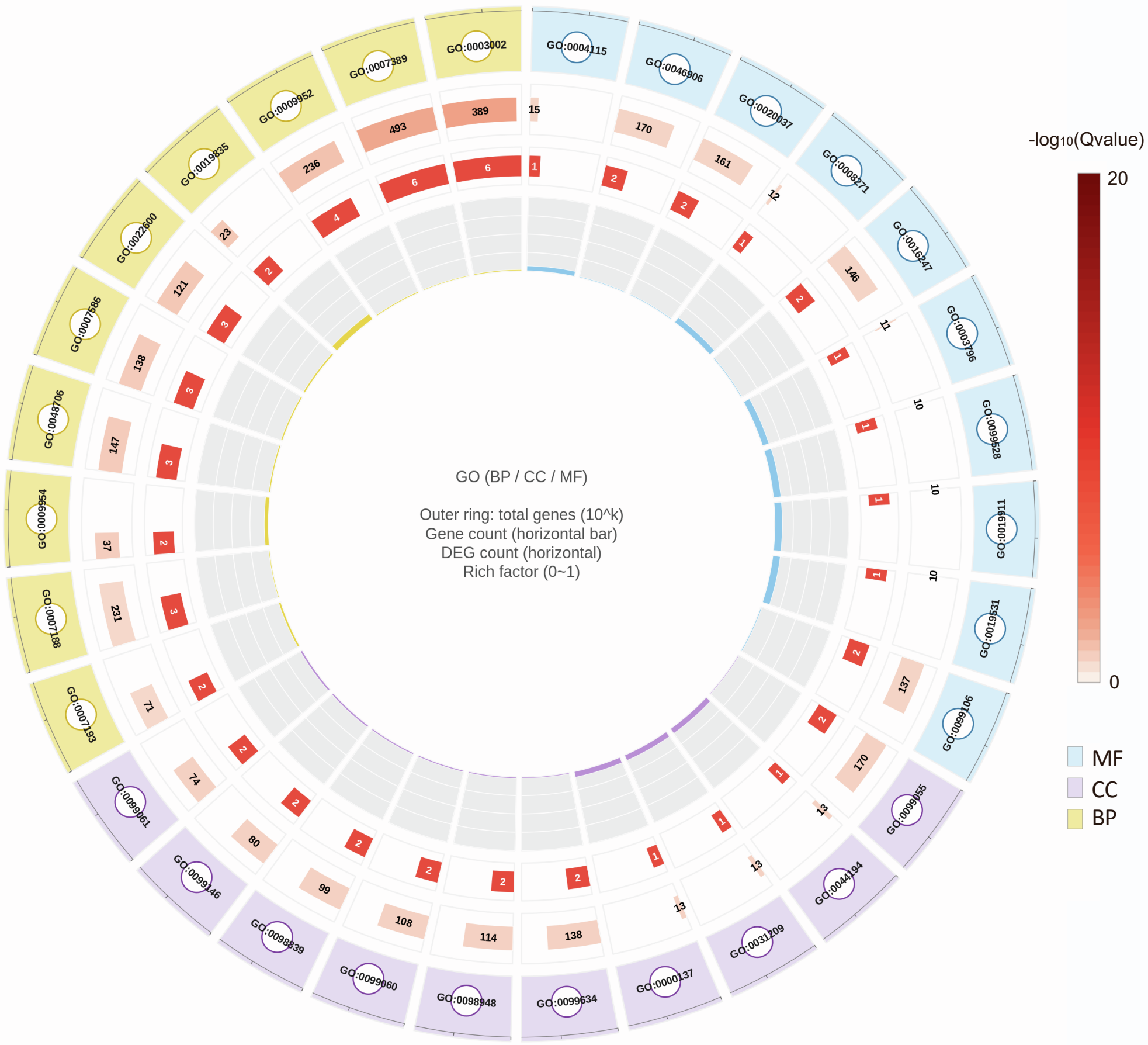

B

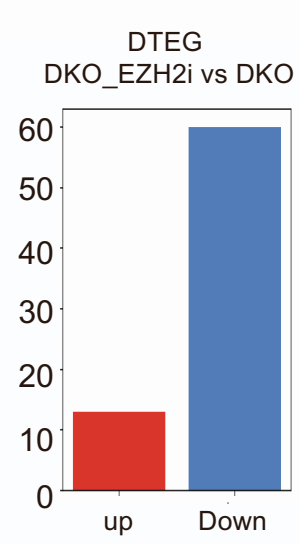

C

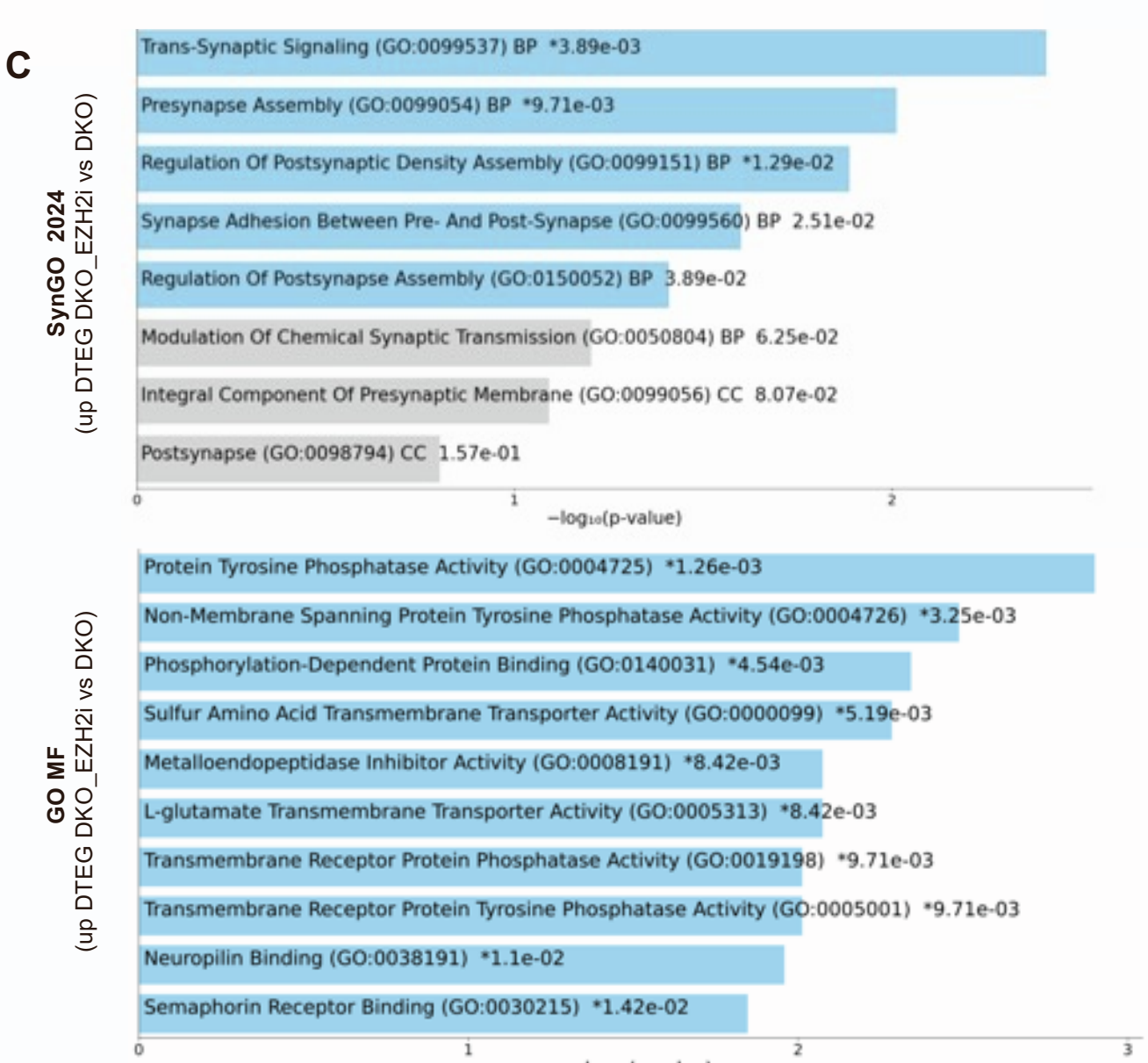

Supplementary Figure 5:
A. Gene Ontology enrichment of homodirectionally regulated genes following EZH2 inhibition. Circular plot showing Gene Ontology (GO) enrichment analysis of genes exhibiting concordant (homodirectional) regulation between transcriptome and translatome in DKO cells treated with EZH2 inhibitor compared to control. GO terms are grouped by category: Biological Process (BP, yellow), Cellular Component (CC, purple), and Molecular Function (MF, blue). The outer ring represents the total number of genes associated with each GO term. Inner bars indicate gene counts, including the number of differentially expressed genes (DEGs) contributing to each term. Color intensity reflects enrichment significance (−log10 Q-value). The innermost track represents the rich factor (ratio of DEGs to total genes per term), indicating the degree of enrichment. This visualization highlights that homodirectionally regulated genes are distributed across diverse biological functions, with enrichment driven by relatively small gene subsets within each category. **B.** Bar plot representing the statistically significant up and down regulated Differentially Translated Genes (DTEG) in DKO cells treated with or without EZH2 inhibitor. **C.** Bar plot showing SynGO 2024 and Gene Ontology Molecular Function (GO MF) analysis including only significantly upregulated TEs between the indicated conditions. Bars represent the magnitude of differential expression (log2 fold change) with only TEs passing the defined statistical threshold (adjusted p value < 0.05) displayed.

Supplementary Figure 6: EZH2 inhibition enhances the response to PI3K inhibition in DKO spheroids and organoids

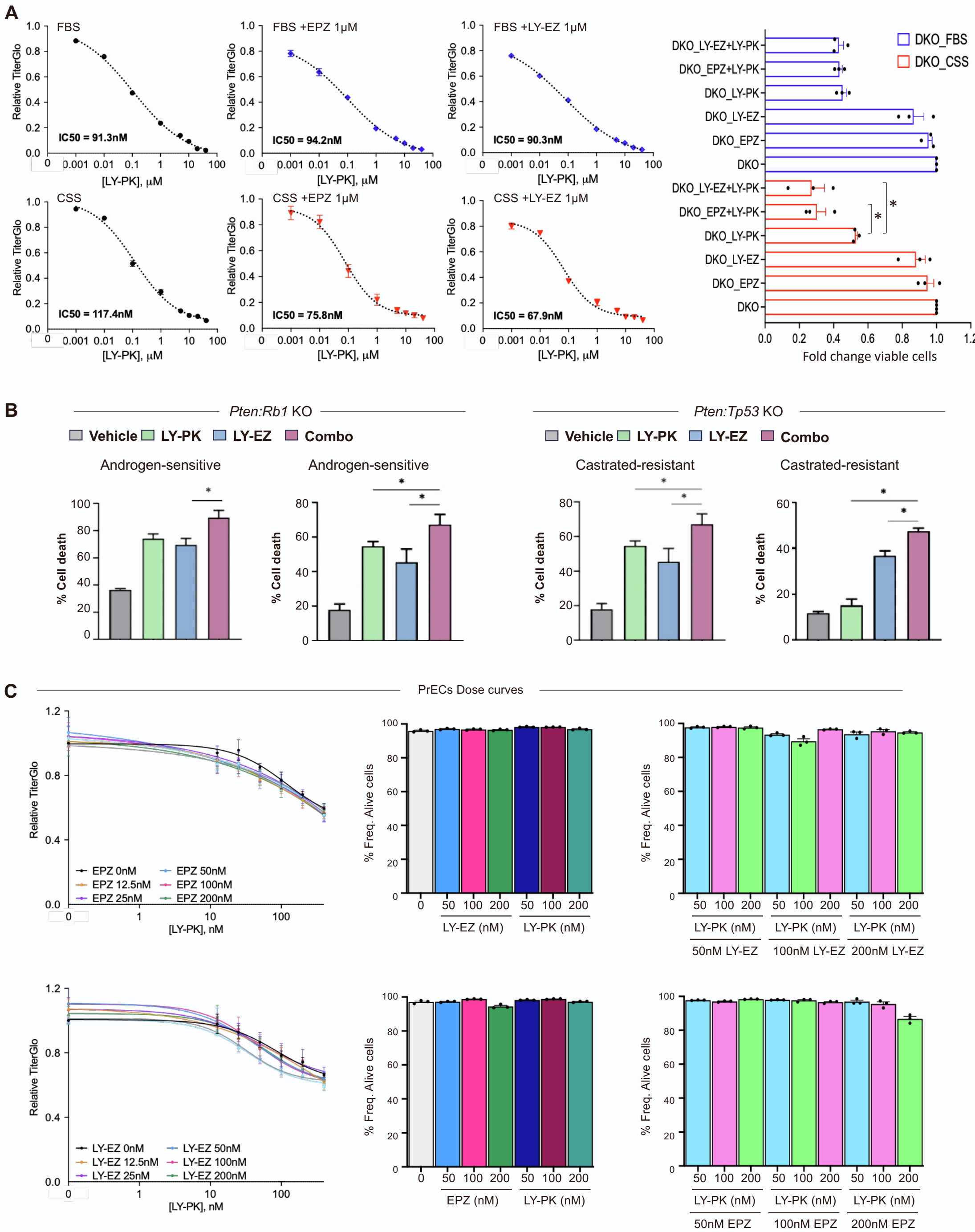

Supplementary figure 6:
A. Relative Titre Glo and cell viability fold change from DKO spheroids cultured in FBS and CSS with a LY-PK dose curve and 1µM of EPZ or LY-EZ. Data represent mean ± SEM from [n=3] independent experiments. * >0.05, ns= no significant (two-tailed Student's t tests was performed). **B.** Bar plot representing the quantification for PI (death) and DAPI (total cell number) in 3D GEMM-derived organoids treated with LY-PK (50nM), LY-EZ (100nM), the combination (Ly-PK+LY-EZ) and with or without Enzalutamide (10µM) for 72 hours. Data represent mean ± SEM from [n=3] independent experiments. *= <0.05, ns= no significant. **C.** Relative Titre Glo curve and bar plots representing propidium iodide staining in PrECs (human primary epithelial cells) cultured in free serum conditions and different doses of LY-EZ or EPZ (from 12.5nM to 200nM). Data represent mean ± SEM from [n=3] independent experiments.

Supplementary figure 7: EZH2 inhibition alters chromatin repression, DNA damage, and proliferation in PPKO and WCM154 tumors

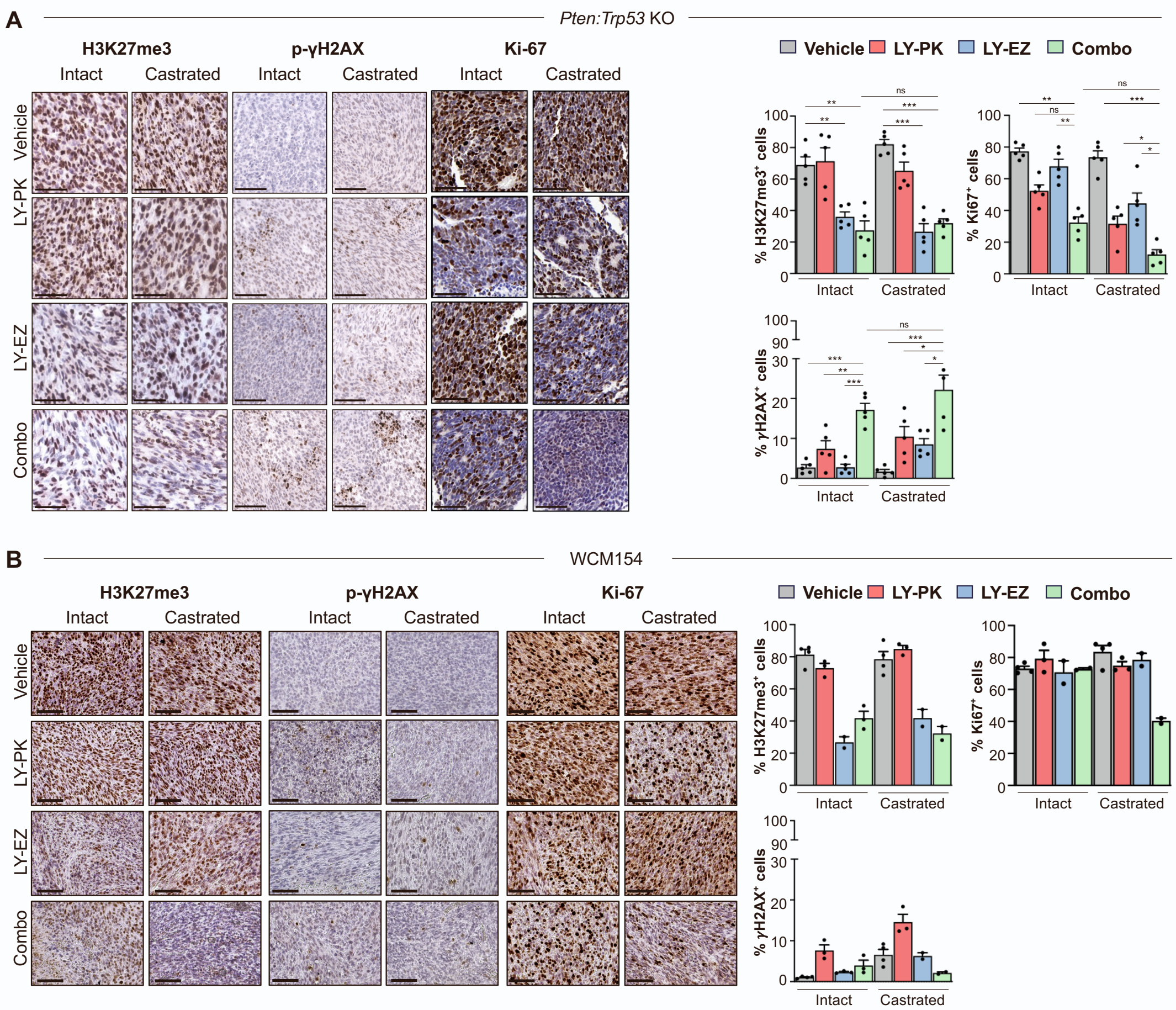

Supplementary Figure 7: A-B H3K27me3, p-γH2AX and Ki-67 immunohistochemistry staining in murine PPKO (*Pten:Trp53* KO) tumors (A) and WCM154 tumors (B), accompanied by the corresponding staining quantification (n = 5 mice per treatment group in PPKO and n=3-2 per treated group in WM154). Data represent mean ± SEM. *= <0.05, **= < 0.01, ***= < 0.001, ns= no significant (two-tailed Student's t tests was performed). Scale bar=50μm.

Supplementary figure 8: Combined LY-PK and LY-EZ treatment is well tolerated in wild-type mice

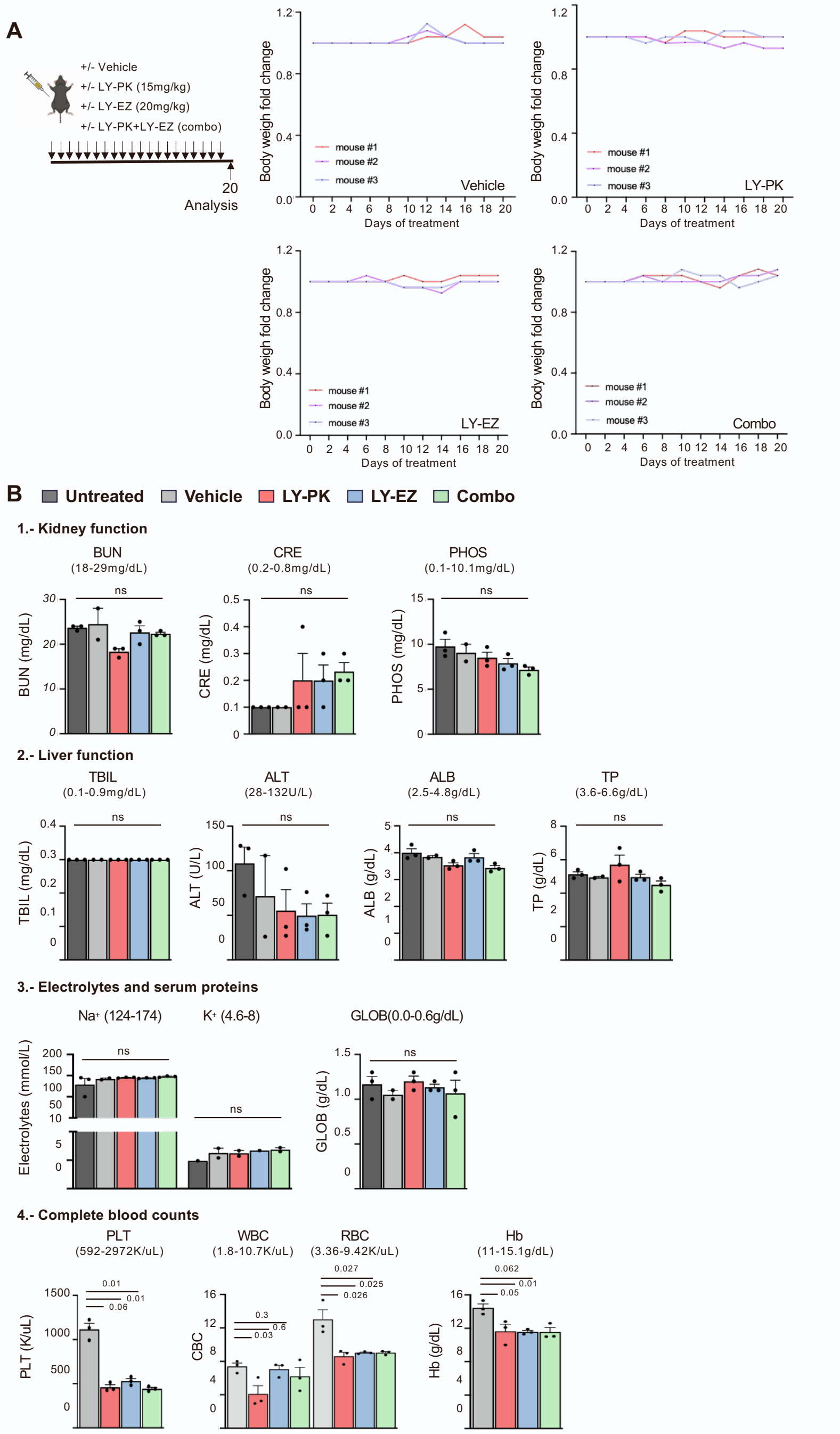

Supplementary figure 8:
A. Schematic representation of the experimental procedure to evaluate the toxicity of the combination therapy in wild type (WT) mice accompanied by the body weight of the mice divided by group of treatment. In brief, C57BL/6N mice were treated daily with LY-PK (15 mg/kg) and/or LY-EZ (20 mg/kg) by intraperitoneal (IP) injection. Body weight was measured every 2 days. (n=3 mice per treatment group (± 1SEM). **B.** Bar plots summarize systemic toxicity parameters measured from serum collected at endpoint including: (1) Kidney function markers (blood urea nitrogen (BEN), creatinine (CRE), and phosphorus (PHOS), (2) Liver function panel (total bilirubin (TBIL), alanine aminotransferase (ALT), albumin (ALB), and total protein (TP), (3) Electrolytes and serum proteins (sodium (Na+), potassium (K+), and globulin (Glob), (4) complete blood counts (platelet count (PLT), white blood cells (WBC), red blood cells (RBC), and hemoglobin (Hb). Data represent mean ± SEM from [n=3 mice]. ns= no significant.

Supplementary Figure 9: EZH2 inhibition alters androgen receptor signaling in DKO and PPKO cells

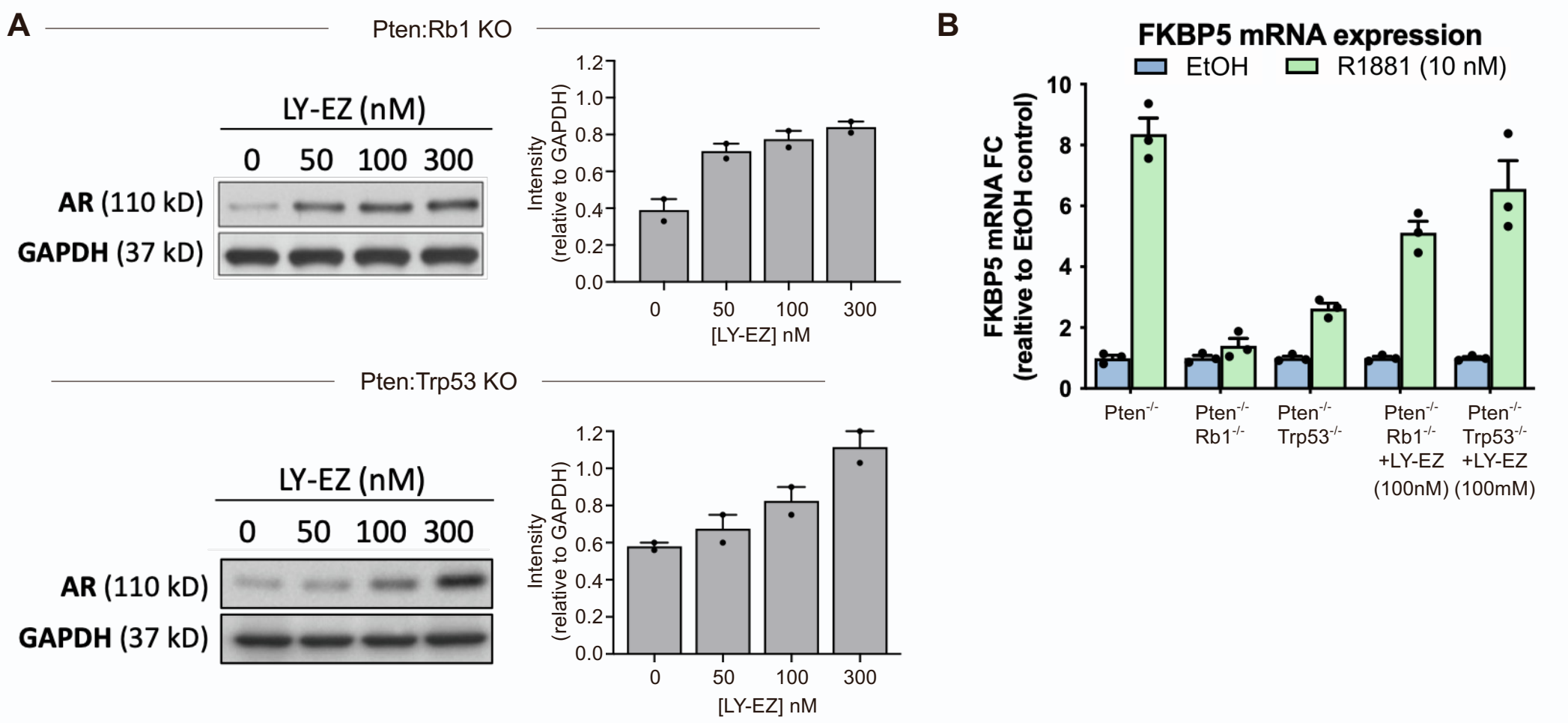

Supplementary figure 9:

A. Western blot indicating AR protein levels and quantification in the DKO and PPKO cells treated with DMSO or LY-EZ at different concentrations (indicated in the figure). **B.** Fold change of Fkbp5 in GEMM-derived DKO and PPKO cells treated with EtOH or R1881 (10 nM) stimulation for 24hrs, in triplicates.

Supplementary Figure 10: Zfp36/TTP associated EZH2 activity to transcriptional programs in DKO and PPKO cells

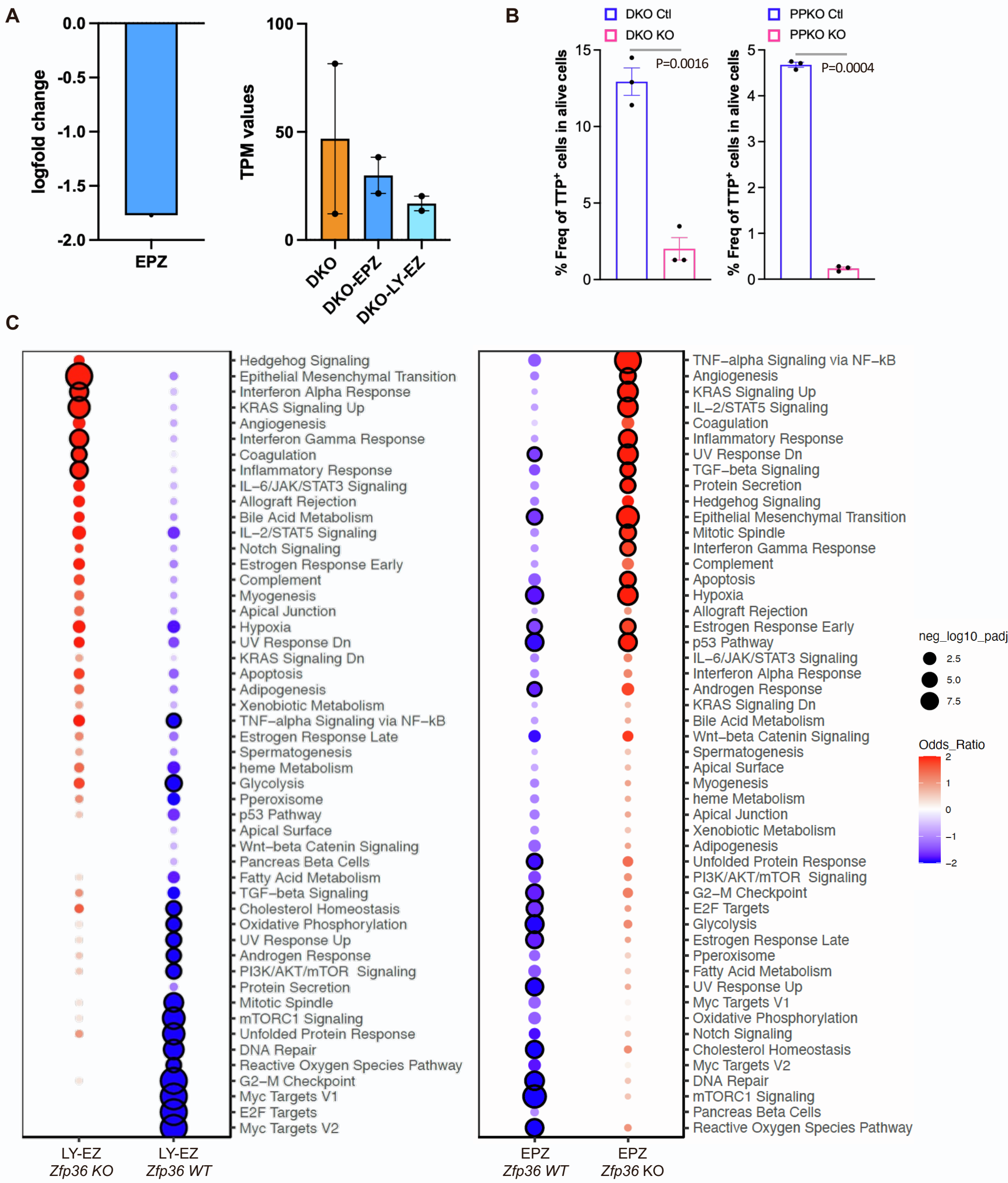

Supplementary figure 10:
A. Bar plots showing the *Zfp36* gene expression in EPZ versus vehicle treated tumors and the *Zfp36* gene expression in our bulk RNAseq in DKO:*Zfp36* KO cells with vehicle or EPZ treatment. **B.** Bar plot representing the TTP flow cytometry analysis in DKO Ctl, PPKO Ctl, DKO *Zfp36* KO, PPKO *Zfp36* KO. **C.** Extended bubble plot demonstrating GSEA analysis of indicated gene signatures from RNAseq derived from DKO and cells treated with DMSO controls or the EZH2i (EPZ6438 or LY-EZ, 5□M) for 96 hours. Hue of red indicates average normalized gene signature level and size of dot indicates percentage of nonzero gene signature.
